# Enforced mesenchymal stem cell tissue colonization counteracts immunopathology

**DOI:** 10.1038/s41536-022-00258-z

**Published:** 2022-10-19

**Authors:** David García-Bernal, Miguel Blanquer, Carlos M. Martínez, Ana I. García-Guillén, Ana M. García-Hernández, M. Carmen Algueró, Rosa Yáñez, María L. Lamana, Jose M. Moraleda, Robert Sackstein

**Affiliations:** 1 Hematopoietic Transplant and Cellular Therapy Unit, Instituto Murciano de Investigación Biosanitaria (IMIB)-Arrixaca, Murcia, Spain; 2 Biochemistry, Molecular Biology and Immunology Department, Faculty of Medicine, University of Murcia, Murcia, Spain; 3 Medicine Department, Faculty of Medicine, University of Murcia, Murcia, Spain; 4 Experimental Pathology Unit, Instituto Murciano de Investigación Biosanitaria (IMIB)-Arrixaca, Murcia, Spain; 5 Exercise Physiology Department, Faculty of Health Sciences, San Antonio Catholic University of Murcia (UCAM), Murcia, Spain; 6 Hematopoietic Innovative Therapies Division, Centro de Investigaciones Energéticas, Medioambientales y Tecnológicas (CIEMAT) and Centro de Investigación Biomédica en Red de Enfermedades Raras (CIBER-ER), Madrid, Spain; 7 Advanced Therapies Mixed Unit, Instituto de Investigación Sanitaria-Fundación Jiménez Díaz (IIS-FJD), Madrid, Spain; 8 Department of Translational Medicine, and the Translational Glycobiology Institute, Herbert Wertheim College of Medicine, Florida International University, Miami, FL USA

**Keywords:** Mesenchymal stem cells, Stem-cell research

## Abstract

Mesenchymal stem/stromal cells (MSCs) are distributed within all tissues of the body. Though best known for generating connective tissue and bone, these cells also display immunoregulatory properties. A greater understanding of MSC cell biology is urgently needed because culture-expanded MSCs are increasingly being used in treatment of inflammatory conditions, especially life-threatening immune diseases. While studies in vitro provide abundant evidence of their immunomodulatory capacity, it is unknown whether tissue colonization of MSCs is critical to their ability to dampen/counteract evolving immunopathology in vivo. To address this question, we employed a murine model of fulminant immune-mediated inflammation, acute graft-versus-host disease (aGvHD), provoked by donor splenocyte-enriched full MHC-mismatched hematopoietic stem cell transplant. aGvHD induced the expression of E-selectin within lesional endothelial beds, and tissue-specific recruitment of systemically administered host-derived MSCs was achieved by enforced expression of HCELL, a CD44 glycoform that is a potent E-selectin ligand. Compared to mice receiving HCELL^−^ MSCs, recipients of HCELL^+^ MSCs had increased MSC intercalation within aGvHD-affected site(s), decreased leukocyte infiltrates, lower systemic inflammatory cytokine levels, superior tissue preservation, and markedly improved survival. Mechanistic studies reveal that ligation of HCELL/CD44 on the MSC surface markedly potentiates MSC immunomodulatory activity by inducing MSC secretion of a variety of potent immunoregulatory molecules, including IL-10. These findings indicate that MSCs counteract immunopathology in situ, and highlight a role for CD44 engagement in unleashing MSC immunobiologic properties that maintain/establish tissue immunohomeostasis.

## Introduction

The most challenging task of the immune system is not so much in mounting a reaction to a given antigen, it is in not reacting so excessively such as to engender fulminant immunopathology. To avert life-threatening tissue destruction within an immunologic “battlefield”, immunoreactivity must be balanced by coincident immunoquiescence, thereby re-establishing immunohomeostasis. Ideally, an effector cell of immunohomeostasis would be natively embedded within the affected tissue(s), poised to locally dampen excessive inflammatory reactions without triggering systemic immunosuppression.

Mesenchymal stem/stromal cells (MSCs)^1,2^ are adult stem/progenitor cells distributed in every organ and tissue of the body. In adult mammals, these cells are most accessible from adipose tissue and bone marrow sources, but can also be readily harnessed from other tissues such as umbilical cord and dental pulp. Though these cells are best known for being progenitors of osteocytes, adipocytes, and chondrocytes, abundant in vitro studies indicate that culture-expanded MSCs possess potent immunomodulatory properties^3–5^. MSC immunoregulatory activity is mediated through a variety of secreted molecules (e.g., transforming growth factor-β (TGFβ), indoleamine 2, 3-dioxygenase (IDO), nitric oxide (NO), and prostaglandin E_2_ (PGE_2_)) and through direct cell–cell contacts^2,6–10^. These properties raise the hypothesis that tissue integrity in the face of florid immunoreactivity could be maintained if sufficient amounts/density of MSCs were present in situ (i.e., within affected tissue(s)) to dampen life-threatening effects of inflammatory effectors. However, despite the fact that preclinical and clinical investigations provide compelling evidence that MSCs mediate immunomodulation^9–15^, we lack knowledge on whether MSC tissue infiltration is key to this effect and whether microenvironmental components trigger this MSC biology. A major hurdle to gaining this knowledge is that MSCs lack molecular effectors of cell migration. As such, when systemically administered, culture-expanded MSCs do not efficiently extravasate at inflammatory sites, and thereby cannot anatomically localize where needed^16^.

Extravasation of circulating cells is critically dependent on their ability to adhere to vascular endothelial cells with sufficient strength to overcome the shear forces of hemodynamic flow. This process is principally regulated by a family of lectins known as “selectins” that bind to a tetrasaccharide determinant known as “sialylated Lewis X” (sLe^X^; CD15s). This structure is comprised of a terminal type 2 lactosamine (i.e., galactose (Gal) β(1,4)-linked to N-acetylglucosamine (GlcNAc)), bearing sialic acid (NeuAc) and fucose (Fuc) substitutions: NeuAc-α(2,3)-Gal-β(1,4)-[Fuc-α(1,3)]-GlcNAc-α1-R. MSCs natively lack of display of sLe^X^, and thus do not express ligands for the endothelial selectin “E-selectin” (CD62E)^17^. However, MSCs uniformly express CD44, a glycoprotein best known for being the principal receptor for hyaluronic acid (HA)^18^. Notably, MSC CD44 is decorated with terminal sialylated type 2 lactosamines^19^, lacking only the presence of fucose in α(1,3)-linkage to GlcNAc to complete the creation of the sLe^x^ determinant. CD44 that is decorated with sLe^X^ is known as “hematopoietic cell E-/L-selectin ligand” (HCELL)^19^, a CD44 glycovariant that is a highly potent E-selectin ligand. Thus, α(1,3)-exofucosylation of MSC CD44 enforces HCELL expression, programming MSC migration to E-selectin-bearing endothelial beds^20^. Importantly, MSCs characteristically display the β1 integrin VLA-4, and engagement of HCELL with vascular E-selectin results in direct activation of VLA-4 in absence of chemokine signaling; subsequent binding of activated VLA-4 to its endothelial ligand, VCAM-1, leads to firm arrest and extravasation^21^. Since E-selectin and VCAM-1 expression are each induced by pro-inflammatory cytokines TNF-α and IL-1, and thereby, both molecules are consistently found in endothelial beds at sites of immunopathology^22,23^, blood-borne cells expressing both HCELL and VLA-4 are primed to home to inflammatory sites.

To examine whether MSC colonization within lesional sites of incipient immune-mediated tissue destruction engenders immunomodulation, we employed a highly reproducible murine model of florid immunopathology: donor splenocyte-enriched full-MHC-mismatched allogeneic hematopoietic stem cell transplantation (allo-HSCT/S) to induce lethal acute graft-versus-host disease (aGvHD). We analyzed the impact of early post-transplant systemic administration of unmodified host-type murine AdMSCs (“UmAdMSCs”, i.e., HCELL^−^ mAdMSCs) and of fucosylated host-type AdMSCs (“FucmAdMSCs”, i.e., HCELL^+^ mAdMSCs) on tissue immunopathology and host survival. Our findings indicate that administration of HCELL^+^ mAdMSCs results in targeted recruitment of mAdMSCs to lesional tissues in aGvHD, with commensurate dampening of inflammatory infiltrates, reversal of the ratios of serum pro-inflammatory: anti-inflammatory cytokines, and deterrence/prevention of immunopathology with resultant improved survival. Mechanistic studies reveal that ligation of surface CD44 of either murine or human MSCs, either via HCELL binding to E-selectin or via CD44 binding to its native ligand hyaluronic acid (HA), potentiates MSC immunomodulation by triggering MSC secretion of multiple immunosuppressive molecules. These findings unveil the cellular biology of tissue-colonizing MSCs as key effectors of immunohomeostasis, indicate that CD44 engagement unleashes MSC immunoregulatory activity, and provide fundamental new insights into how the pathophysiologic display of endothelial E-selectin display can be leveraged to prevent and/or reverse immunopathology.

## Results

### E-selectin expression is upregulated in microvessels within target tissues of aGvHD

**Fig. 1:**
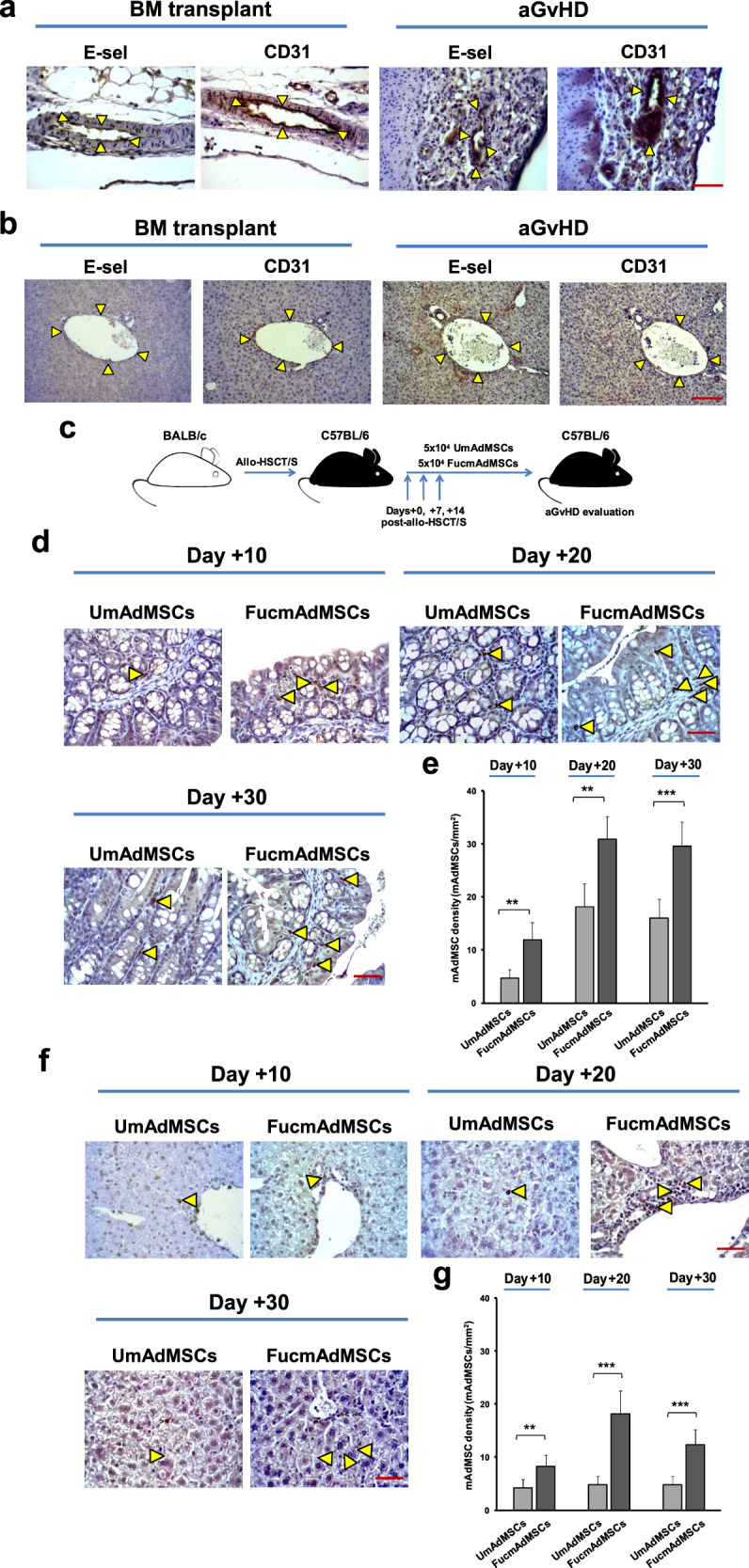
FTVII treatment significantly increases mAdMSC colonization within aGvHD-target organs. **a** Immunohistochemical staining of sequential sections of C57BL/6 intestines, or **b** livers showing co-localization of E-selectin and endothelial marker CD31 (delimited by arrowheads) in mice with ongoing aGvHD compared to bone marrow transplanted mice (no aGvHD) (magnification ×400, Scale bar: 50 μm) (*n* = 5 mice per group). Results are representative of *n* = 5 separate experiments. **c** Schematic of the experimental protocol for mAdMSCs administration after allo-HSCT/S. **d** Sections of small intestines, or **f** livers are shown from recipient C57BL/6 mice with ongoing aGvHD that were administered GFP-transgenic-mAdMSCs either unmodified (UmAdMSCs) or FTVII-modified (FucmAdMSCs). Sections were stained for expression of GFP by anti-GFP ABC colorimetric immunohistochemistry. Representative images of small intestines and livers from both groups of treated animals at days 10, 20, and 30 post-transplant show infiltrating GFP^+^ mAdMSCs (magnification ×200, Scale bar: 100 μm). Arrowheads indicate infiltrating GFP^+^ mAdMSCs in intestinal lamina propria or in hepatic portal area, respectively. Bar graphs represent absolute GFP^+^ mAdMSC infiltrate counts in **e** small intestines, or **g** livers as mean ± SD from counts relative to 1 mm^2^ (*n* = 5 mice per group). GFP^+^ mAdMSCs infiltration was significantly increased in recipients of FucmAdMSCs compared to that of UmAdMSCs, **p < 0.01 or ***p < 0.001, res*p*ectively, analyzed by one-way ANOVA with Tukey’s multiple-comparisons test.

Vascular E-selectin expression was assessed by immunohistochemistry in samples of skin, liver, and intestine from healthy C57BL/6 mice (H-2^b^), and from C57BL/6 mice that were transplanted with MHC-mismatched BALB/c (H-2^d^) bone marrow with donor splenocytes (“allo-HSCT/S” group) or without splenocytes (“allo-HSCT” group). In mice without aGvHD (healthy C57BL/6 mice and those allo-HSCT mice receiving “bone marrow only”, i.e., without addition of donor splenocytes), no significant E-selectin staining was observed in skin, liver or intestinal vessels. However, in allo-HSCT/S mice (all of whom developed florid aGvHD and died within 14 days post-transplant), microvessels in the intestine (Fig. 1a), liver (Fig. 1b), and skin (Supplementary Fig. 1) consistently displayed E-selectin, which co-localized with the endothelial marker CD31 in sequential sections. Notably, the intensity of E-selectin expression in gut and liver was uniformly higher than that in skin.

### Systemically-administered HCELL^+^ mAdMSC colonize tissues affected by aGvHD but not lymphoid organs

As with culture-expanded murine bone marrow-derived MSCs, culture-expanded mAdMSCs natively lack expression of sLe^x^, and, correspondingly, do not bind to E-selectin^24^. As reported previously, exofucosylation of mAdMSCs using fucosyltransferase VII (FTVII) engenders potent E-selectin binding by installing display of sLe^x^ uniquely on CD44, thereby creating HCELL, and has no effect on the expression of characteristic markers CD29, CD44, CD49d, CD73, CD90, CD105, CD106, and CD166, nor the capacity of cells to differentiate into adipocytes, chondrocytes or osteoblasts^17,24,25^.

To analyze the effect of enforced HCELL expression on mAdMSC tissue colonization, we utilized recipient-type GFP^+^ mAdMSCs to track parenchymal distribution following systemic administration. To this end, allo-HSCT/S mice received 5 × 10^4^ GFP^+^ mAdMSCs, either exofucosylated (FucmAdMSCs) or not (UmAdMSCs), by intravenous injection on days 0, +7, and +14 post-transplant (schematic diagram shown in Fig. 1c). GFP^+^ mAdMSCs were then identified by immunohistochemistry within mesenteric and peripheral lymph nodes, spleen, skin, liver, and gut of recipient mice. FucmAdMSCs and UmAdMSCs were not detectable in any lymphoid tissues or in skin at any time-point post-transplantation. However, as early as day 10 post-HSCT (day + 10), exofucosylated mAdMSC showed marked intestinal tropism, with FucmAdMSC infiltrates in intestinal lamina propria being three-fold higher than that of mice receiving UmAdMSCs. Intestinal infiltrates steadily increased, plateauing at day +20 post-HSCT (Fig. 1d, e). Similarly, histological analysis of livers obtained at days +10, +20, and +30 from animals treated with FucmAdMSCs showed significantly higher numbers of GFP^+^ cells in hepatic periportal areas compared to that of mice receiving UmAdMSCs (Fig. 1f, g).

### Enforced mAdMSC colonization within aGvHD target site improves survival, reduces both clinical and histopathologic severity of aGvHD, and significantly decreases T cell and neutrophil lesional infiltrates

**Fig. 2:**
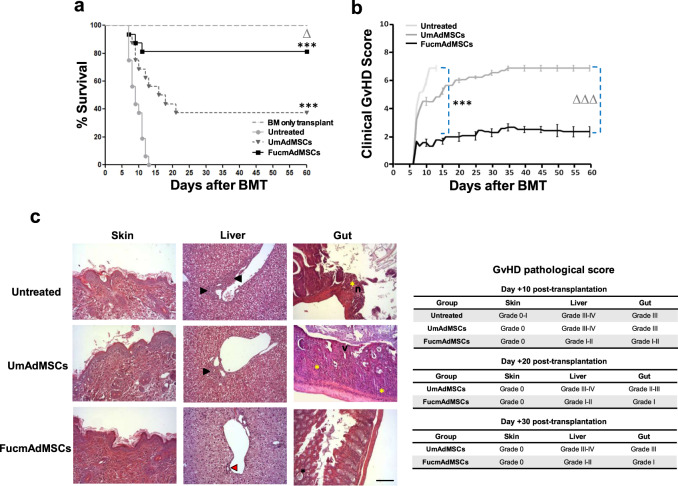
Allo-HSCT/S recipients administered HCELL^+^ mAdMSCs have improved survival, improved aGvHD scores, and significantly reduced aGvHD-associated tissue damage. C57BL/6 recipient mice were transplanted intravenously via tail vein with 1 × 10^7^ bone marrow cells (“BM only transplant”) or with 1 × 10^7^ bone marrow cells enriched with 1.5 × 10^7^ donor splenocytes to induce aGvHD (“Allo-HSCT/S”). On day +0, +7, and +14 allo-HSCT/S recipient mice received an intravenous infusion of 5 × 10^4^ mAdMSCs, either unmodified (“UmAdMSCs” mice) or FTVII-modified mAdMSCs (“FucmAdMSCs” mice). At the same time periods, another group of allo-HSCT/S mice received an equal volume of saline solution (“Untreated” mice). **a** Kaplan–Meier survival curves of recipient C57BL/6 of the different groups are shown (*n* = 16 animals per group). Survival in all-HSCT/S mice administered HCELL^+^ mAdMSCs (“FucmAdMSCs”) was significantly higher compared to the allo-HSCT/S “Untreated” (i.e., no administration of mAdMSCs) group (***p < 0.001) or compared to allo-HSCT/S mice receiving UmAdMSCs (^Δ^*p* < 0.05), analyzed by Log-rank (Mantel–Cox) test. **b** Clinical aGvHD score for each experimental group was assessed using a composite scoring system consisting of 5 clinical individual scores (weight loss, posture, activity, fur texture, and skin integrity (maximum index = 10)). Clinical aGvHD score in allo-HSCT/S mice administered HCELL^+^ mAdMSCs (“FucmAdMSCs”) was significantly reduced compared to “Untreated” group of animals (***p < 0.001) or compared to mice administered UmAdMSCs (^ΔΔΔ^*p* < 0.001), analyzed by one-way ANOVA with Tukey’s multiple-comparisons test. **c** Histopathological aGvHD score was evaluated in distant areas of skin, liver, and gut of mice from the different experimental groups at day +10 post-transplantation by hematoxylin and eosin (H&E) staining. In skin, no significant histopathology lesions could be identified. In liver, inflammatory infiltrate could be identified surrounding >50% of bile ducts (black arrowheads) in untreated and UmAdMSCs-treated groups, whereas in livers from FucmAdMSCs-treated animals, 75–50% of bile ducts (red arrowhead) were unaffected. Regarding the gut, while moderate villous atrophy (v), focal mucosal ulceration (n), and inflammatory infiltrate (yellow asterisks) could be evidenced in untreated and UmAdMSCs-treated groups, slight inflammatory infiltrate and/or crypt epithelial cells apoptosis (black asterisk) were identified in FucmAdMSCs guts. H&E staining images from the different organs shown (magnification ×200, Scale bar: 100 μm) are representative of *n* = 8 animals per group.

To assess whether enhanced mAdMSC tissue infiltration within aGvHD target sites impacts immune-mediated tissue damage, clinical outcome and tissue histology were evaluated in animals undergoing allo-HSCT and allo-HSCT/S, without or with intravenous infusions of 5 × 10^4^ recipient-type UmAdMSC or FucmAdMSCs on days 0, +7, and +14 post-transplantation. Mice in the allo-HSCT group (i.e., “BM only transplant” group) did not develop aGvHD, whereas mice receiving allo-HSCT/S without mAdMSC administration (“Untreated” group) displayed a rapid development of florid aGvHD, resulting in death in all mice within 14 days post-transplant (Fig. 2a). Allo-HSCT/S mice that received early post-transplant infusions of UmAdMSCs displayed significantly decreased aGvHD-related mortality compared to mice not receiving mAdMSCs (*p* < 0.001), but infusions of FucmAdMSCs yielded a profound survival advantage compared to those receiving UmAdMSCs (82% versus 38% survival, *p* < 0.05). As shown in Fig. 2b, the marked improved survival of the FucmAdMSC-treated group of animals correlated with a sustained reduction of the clinical aGvHD score, being significantly lower than that observed for mice receiving UmAdMSCs or no mAdMSCs (*p* < 0.001).

The GvHD pathologic score was measured by histopathologic grading of aGvHD target organs beginning at day +10. Whereas no observable lesions were noted in the skin of mice at early times post-transplantation, histological analysis of liver and gut revealed significant differences in the severity of aGvHD between the treatment groups. Livers of Untreated and of UmAdMSC-treated animals showed extensive epithelial damage with abundant periportal inflammatory infiltrates and destruction of the majority of bile ducts (grade III–IV aGvHD), whereas FucmAdMSC-treated mice showed only minimal hepatic injury (grade I–II) (Fig. 2c). Moreover, small intestines of Untreated mice or of mice that received UmAdMSCs similarly displayed focal mucosal ulcerations with moderate villous atrophy (grade III), whereas small intestines of mice receiving FucmAdMSCs had only scattered individual apoptotic cells and limited villous atrophy (grade I–II). Significantly, at later times post-transplantation (days +20 and +30), mice receiving FucmAdMSCs showed reduced aGvHD-associated damage in both liver and small intestine compared to their UmAdMSC-treated counterparts (Fig. 2c).

**Fig. 3:**
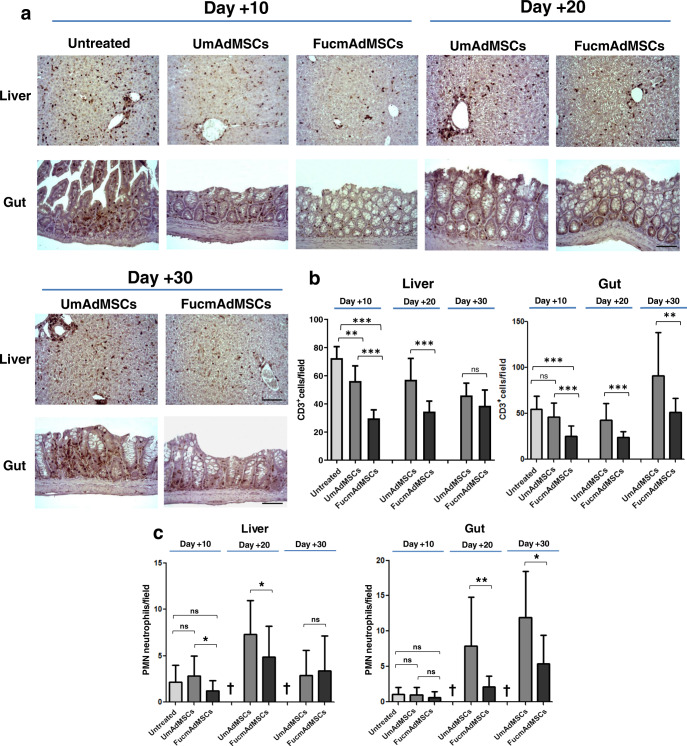
Allo-HSCT/S recipients administered HCELL^+^ mAdMSCs have lower CD3^+^ lymphocyte and polymorphonuclear neutrophil (PMN) infiltrates in liver and gut. Tissues from animals with ongoing aGvHD, either Untreated or treated with either type of mAdMSCs, were isolated after 10, 20, or 30 days after allo-HSCT/S. **a** CD3^+^ T cell inflammatory infiltrate was then detected by standard anti-CD3 ABC colorimetric immunohistochemistry. Representative images of CD3^+^ T cell infiltrates within liver and gut from the different mouse groups are shown (magnification ×200, Scale bar: 100 μm). **b** Bar graphs depict absolute T cells counts (CD3^+^ cells), presented as mean ± SD per high-power field from counts relative to 10 high-power fields (magnification ×200) (*n* = 5 mice per group). Compared to Untreated mice, mice receiving UmAdMSCs and FucmAdMSCs had significantly less T cell infiltrates, with lowest infiltrates in those mice receiving FucmAdMSCs (**p < 0.01 or ***p < 0.001), analyzed by one-way ANOVA with Tukey’s multiple-comparisons test. **c** PMNs were identified in tissue sections on basis of characteristic morphologic appearance of lobulated nuclei. Absolute PMN counts are presented as mean ± SD per high-power field from counts relative to 10 high-power fields (magnification ×400) (*n* = 5 mice per group). As shown by asterisks, statistically significant differences in the extent of PMN infiltrates were observed among mice that received UmAdMSCs versus those that received FucmAdMSCs, *p < 0.05 or **p < 0.01, res*p*ectively, analyzed by one-way ANOVA with Tukey’s multiple-comparisons test.

Given the reported correlation between the amounts of T cell infiltration and the lesional severity of aGvHD-affected organs^26,27^, we analyzed levels of CD3^+^ T cells within skin, liver, and gut of surviving animals at different time-points after allo-HSCT/S. Livers of mice that did not receive mAdMSCs displayed abundant and extensive areas of inflammatory T cell infiltrates within hepatic portal triads at day +10, which were significantly higher than those observed in both mAdMSC-treated groups (*p* < 0.001), with lowest levels observed in FucmAdMSC-treated mice (Fig. 3a, b). Remarkably, after 10–20 days post-HSCT/S, CD3^+^ T cell counts were significantly lower in livers from animals receiving FucmAdMSCs compared to the UmAdMSC-treated group (*p* < 0.001). At longer times post-HSCT (day +30), the T cell burden in livers of surviving UmAdMSC-treated animals significantly decreased, showing levels similar to that observed in the FucmAdMSC-treated group. The same trend of decreasing numbers of infiltrating T cells was also observed in the intestinal lamina propria of surviving mice at days +10 and +20, although UmAdMSC-treated mice displayed a consistently higher number of T cell infiltrates in gut compared to that of FucmAdMSC-treated mice (*p* < 0.01). Interestingly, however, intestinal T cell infiltrates increased between day +20 and day +30 in the mAdMSC-treated mice to levels higher than at days +10 and +20, but infiltrates were consistently less in FucmAdMSC-treated mice compared to UmAdMSC counterparts (Fig. 3a, b).

In addition to levels of T cell infiltrates, increasing quantities of neutrophils in aGvHD-affected organs are highly predictive of disease severity^28^. Thus, we analyzed neutrophil infiltrates in liver and gut in surviving mice that received allo-HSCT/S (Fig. 3c). At day +10, there were scarce neutrophil infiltrates in liver and gut in the Untreated, UmAdMSC-treated and FucmAdMSC-treated mice consistent with expected neutropenia (i.e., pre-engraftment). However, at day +20, neutrophil infiltrates were prominent in liver and gut from UmAdMSC-treated animals, whereas neutrophil infiltrates were sparse in the FucmAdMSC-treated mice, especially in the gut (*p* < 0.01). At day +30 compared to day +20, similar to that observed for infiltrating T cells, surviving mice had lower neutrophil infiltrates in liver, but higher infiltrates in gut. However, consistently, mice receiving FucmAdMSCs displayed lower neutrophil numbers in these organs compared to mice treated with UmAdMSCs. There was a close inverse linear correlation between the increased numbers of intestinal mAdMSCs and the lower burden of T cells and neutrophils in gut of animals receiving FucmAdMSCs at days +20 and +30 post-transplant (Pearson correlation coefficients: −0.574 and −0.897 for T cells and neutrophils, respectively). Importantly, there were no significant differences in peripheral blood leukocyte counts among any of the treatment groups (values ×10^3^/μl, mean +/− SD for each group: (1) Untreated 1.63 +/− 0.21 lymphocytes and 0.24 +/− 0.12 neutrophils; (2) UmAdMSC-treated 1.78 +/− 0.59 lymphocytes and 0.38 +/− 0.17 neutrophils; (3) FucmAdMSC-treated 1.97 +/− 0.80 lymphocytes and 0.30 +/− 0.22 neutrophils), indicating that the observed variances in levels of lymphocyte and neutrophil tissue infiltrates were not due to variations in levels of hematopoietic engraftment.

### FucmAdMSC administration in mice with aGvHD prominently alters plasma levels of pro-inflammatory and anti-inflammatory cytokines

**Fig. 4:**
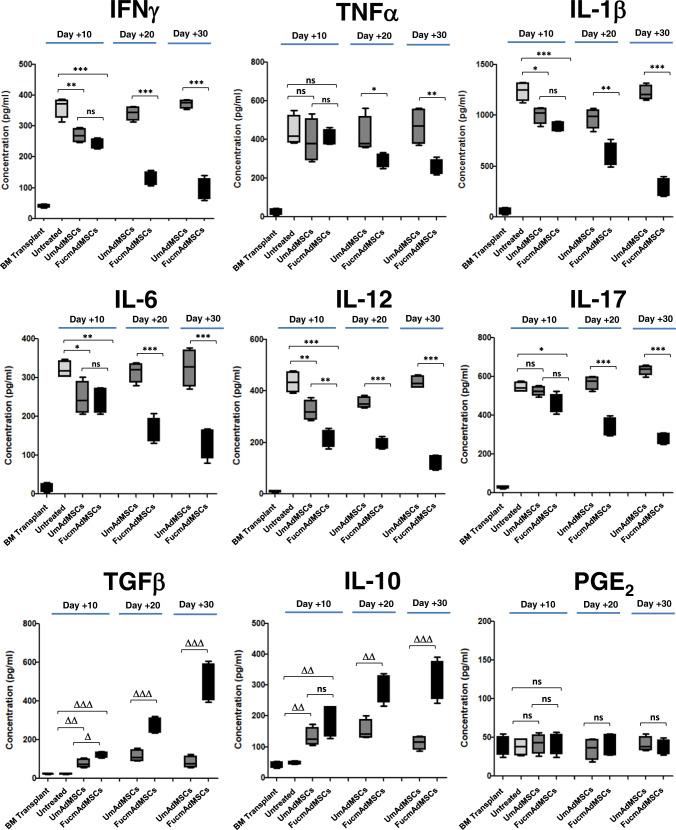
Intravenous infusion of FTVII-treated mAdMSCs alters the systemic profile of secreted pro-inflammatory and anti-inflammatory cytokines on mice with aGvHD. Plasma concentrations of the pro-inflammatory factors IFNγ, TNFα, IL-1β, IL-6, IL-12, and IL-17, and anti-inflammatory molecules TGFβ, IL-10, and PGE_2_ were measured on days +10, +20, or +30 post-transplantation by ELISA. Data are presented as mean ± SD of *n* = 5 animals per group. As shown in the panels, plasma levels of all the inflammatory cytokines tested were significantly decreased with administration of FucmAdMSCs (*p < 0.05, **p < 0.01, ***p < 0.001), whereas levels of anti-inflammatory cytokines TGFβ and IL-10 were markedly increased (^Δ^*p* < 0.05, ^ΔΔ^*p* < 0.01, ^ΔΔΔ^*p* < 0.001), analyzed by one-way ANOVA with Tukey’s multi*p*le-comparisons test.

Apart from profound cellular changes within the tissues, and consistent with results of others^29,30^, we observed that mice receiving allo-HSCT/S without mAdMSC infusion (i.e., Untreated animals) showed marked increases in plasma levels of the pro-inflammatory cytokines IFNγ, TNFα, IL-1β, IL-6, IL-12, and IL-17 on day +10 post-transplantation, and low levels of the anti-inflammatory cytokines TGFβ and IL-10 (Fig. 4). Importantly, administration of UmAdMSCs or FucmAdMSCs yielded a steep decrease in pro-inflammatory mediators compared to that of Untreated mice, with strikingly lower levels of TNFα, IL-1β, IL-6, IL-12, and IL-17 in those mice receiving FucmAdMSCs. Plasma concentrations of PGE_2_, an anti-inflammatory factor released by MSCs^8^, did not change among any of the treatment groups, but mice receiving mAdMSCs had increased levels of anti-inflammatory cytokines TGFβ and IL-10, with markedly higher levels of these cytokines in those mice receiving FucmAdMSCs. Thus, as compared to mice receiving UmAdMSCs, FucmAdMSC-treated mice displayed a more persistent and more profound depression of plasma levels of pro-inflammatory cytokines with much higher and more sustained increases in anti-inflammatory cytokines.

### HCELL/CD44 ligation by either E-selectin or HA, respectively, augments mAdMSC-induced inhibition of mitogen-stimulated splenocyte proliferation and boosts production of immunoregulatory molecules by both murine and human MSCs

**Fig. 5:**
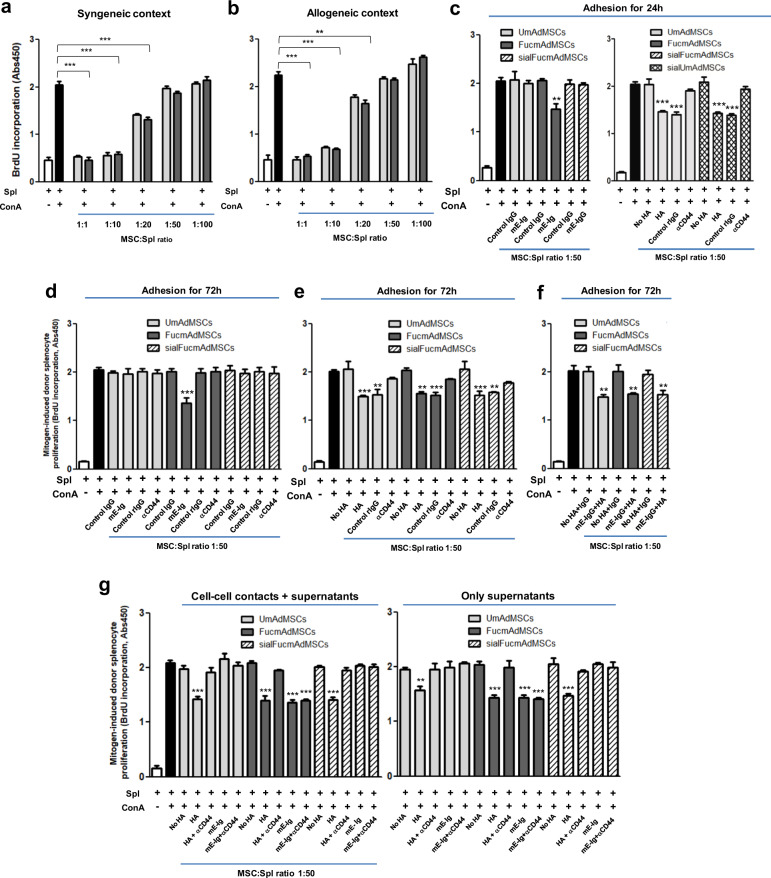
Effects of HCELL/CD44 engagement on immunomodulatory properties of mAdMSCs. **a** Splenocytes (Spl) from C57BL/6 (syngeneic context) or **b** from BALB/C mice (allogeneic context) were stimulated with concanavalin A (ConA) in the presence of different ratios of unmodified (UmAdMSCs) or FTVII-modified AdMSCs (FucmAdMSCs) from C57BL/6 mice. Mitogen-induced proliferation of responder splenocytes was measured by incorporation of BrdU. In presence of mAdMSCs, proliferation of responder splenocytes was significantly inhibited (**p < 0.01 or ***p < 0.001). **c**–**f** BALB/C splenocytes were stimulated with ConA in the presence of UmAdMSCs, FucmAdMSCs, or sialidase-treated UmAdMSCs (sialUmAdMSCs) all derived from C57BL/6 mice) at an MSC:splenocyte ratio of 1:50 (allogeneic context) that were previously cultured for **c** 24 h with E-selectin (mE-Ig) (left) or hyaluronic acid (HA) (right), or **d** previously cultured for 72 h with mE-Ig, **e** HA, or **f** both ligands, and then maintained in wells containing mE-Ig, HA, or both, respectively. As controls, E-selectin adherence of FucmAdMSCs was abrogated by sialidase treatment of the mAdMSCs (sialFucmAdMSCs), or by blocking adherence of mAdMSC to HA by culturing in presence of a blocking anti-CD44 antibody. Responder splenocyte proliferation was significantly inhibited (*p < 0.05, **p < 0.01, or ***p < 0.001), respectively. **g** Inhibition of splenocyte proliferation in presence of conditioned media obtained from HA- or mE-Ig-ligated UmAdMSCs, FucmAdMSCs, or sialFucmAdMSCs (right) was analyzed compared to levels obtained in the continuous presence of the same type of mAdMSCs (left). As shown in panel at right, proliferation was significantly inhibited in presence of supernatant alone (***p < 0.001). Data were analyzed by one-way ANOVA with Tukey’s multiple-comparisons test.

To analyze whether exofucosylation itself impacts the immunoregulatory properties of mAdMSCs, we evaluated the capacity of UmAdMSCs and FucmAdMSCs to suppress mitogen-induced proliferation of both syngeneic and allogeneic murine T cells. To this end, responding splenocytes were stimulated with concanavalin A (ConA) in the presence of varying amounts of both types of mAdMSCs, from ratios of 1:1 to 1:100 MSC:splenocyte. Co-incubation with UmAdMSCs or FucmAdMSCs significantly inhibited mitogen-stimulated splenocyte proliferation in a dose-dependent manner from ratio 1:1 to ratio 1:20, with identical inhibitory effects using both types of mAdMSCs, in both syngeneic (Fig. 5a) and allogeneic contexts (Fig. 5b). These findings indicate that mAdMSC-mediated suppression of mitogen-induced T cell proliferation is unaffected by the exofucosylation process and the resulting fucose installation on CD44.

The observed higher MLR-suppressive effect coincident with increased ratios of MSCs:splenocytes suggests that augmentation of MSC colonization in aGvHD-affected tissues (as was observed following the administration of FucmAdMSCs (see Fig. 1e, g)) could, alone, contribute to ameliorating inflammation. However, apart from increased MSC colonization, we also reasoned that since HCELL^+^ MSCs engage E-selectin during the process of extravasation, HCELL ligation could impact the immunobiology of FucmAdMSCs. Accordingly, we performed in vitro mitogenic assays of mAdMSC/ConA-stimulated splenocyte (“ConA-splenocyte”) co-cultures using mAdMSCs that were incubated with mE-Ig chimera or isotype control human IgG1 for 24 h prior to introduction of splenocytes, and contact with mE-Ig chimera or IgG1, respectively, was maintained during the co-culture period. As shown in Fig. 5c (left), at 1:50 ratio of MSC:ConA-splenocyte in the mitogenic assay, FucmAdMSCs (i.e., HCELL^+^ mAdMSCs) previously incubated for 24 h with mE-Ig, but not with isotype control IgG1, more completely dampened donor T cell proliferation than FucmAdMSCs not exposed to mE-Ig. Importantly, this effect was abrogated by elimination of E-selectin adherence by sialidase treatment of FucmAdMSCs (“sialFucmAdMSCs”, which are HCELL^−^), indicating that the observed augmented anti-proliferative effect is directly related to the capacity of HCELL to engage E-selectin. To determine whether CD44 ligation itself triggers this immunomodulatory effect, we performed mitogenic assays using UmAdMSCs previously cultured for 24 h in presence of the conventional CD44 ligand, HA, with continued exposure to HA during the co-culture with ConA-splenocytes. As is shown in Fig. 5c (right), ligation of CD44 with HA induced a similar anti-proliferative effect, and, consistent with specificity for engagement of CD44, there was no boosting of UmAdMSC (i.e., HCELL^−^ mAdMSC) anti-mitogenic activity in presence of mE-Ig.

To further evaluate the effects of E-selectin-mediated HCELL ligation or HA-mediated CD44 ligation in MSC immunobiology, we performed in vitro mitogenic assays using ConA-splenocytes and HCELL^+^ mAdMSCs or HCELL^−^ mAdMSCs that were previously incubated for 72 h in presence or absence of input E-selectin and HA (Fig. 5d–f). Extending the E-selectin or HA pre-incubation time from 24 to 72 h did not further potentiate the relevant MSC-mediated anti-proliferative effect. However, whereas UmAdMSCs or sialFucmAdMSCs (each being HCELL^−^ mAdMSCs) displayed an improved anti-mitogenic effect only after CD44-mediated HA ligation (an effect that was abrogated in presence of a blocking anti-mouse CD44 antibody) (Fig. 5e), FucmAdMSCs exhibited a substantial anti-mitogenic effect after either HA or E-selectin engagement that was abrogated by function-blocking anti-CD44 mAb treatment or sialidase treatment, respectively (Fig. 5d, e); the fact that HA exposure of MSCs equally enhanced the anti-mitogenic effect of FucmAdMSCs and UmAdMSCs indicates that the creation of HCELL by exofucosylation does not affect the capacity of CD44 to bind HA, and, thus, HCELL engagement of either HA or E-selectin potentiates MSC immunomodulation. Importantly, for either UmAdMSCs or FucmAdMSCs, the simultaneous pre-incubation with both ligands (i.e., mE-Ig and HA) did not additively augment the MSC immunomodulatory effect on lymphocyte proliferation (Fig. 5f), and, moreover, sialidase-treated FucmAdMSCs (sialFucmAdMSCs) and sialidase-treated UmAdMSCs (sialUmAdMSCs) did not show an increased anti-mitogenic effect after HA-mediated ligation of CD44 compared to HA-ligation of FucmAdMSCs or UmAdMSCs not treated with sialidase (Fig. 5c, right). This latter result indicates that, in contrast to reported findings in other cell types^31^, the native sialylation of CD44 (on terminal type 2 lactosamines) of MSCs does not inhibit its binding to HA. Altogether, these results indicate that ligation of the CD44 protein, whether as HCELL via E-selectin or CD44/HCELL via HA, unleashes MSC anti-mitogenic effects.

**Fig. 6:**
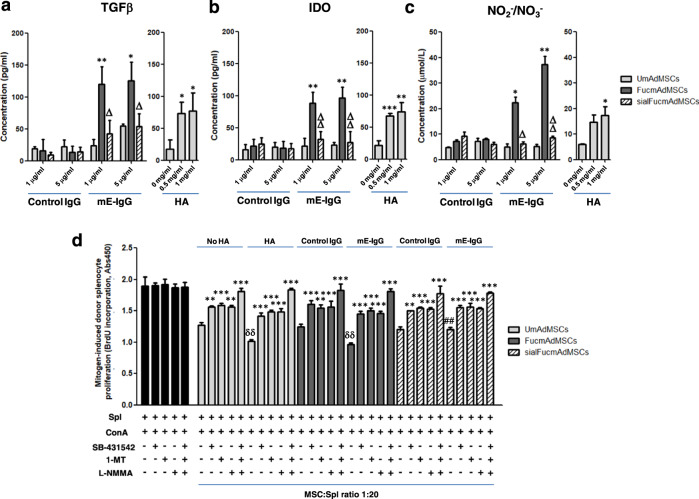
Levels of TGFβ, IDO, and of NO metabolites in supernatants of mAdMSCs after HCELL or CD44 ligation. UmAdMSCs or FucmAdMSCs were cultured in the presence of different concentrations of E-selectin (mE-Ig) or hyaluronic acid (HA) at 37 °C for 24 h, and culture supernatants were then collected. **a**–**c** Levels of anti-inflammatory molecules **a** TGFβ, **b** IDO, and **c** NO metabolites (e.g., NO_2_^−^/NO_3_^−^) were measured by ELISA techniques. Level of each molecule was significantly increased by ligation of CD44 or of HCELL as shown (*p < 0.05 or **p < 0.01); treatment of FucmAdMSCs with sialidase (“sialFucmAdMSCs”) abrogated HCELL ligation with mE-Ig, with commensurate decreased levels of anti-inflammatory molecules compared to levels found in FucmAdMSCs cultures exposed to E-selectin (^Δ^*p* < 0.05 or ^ΔΔ^*p* < 0.01). **d** Inhibitory agents to each molecule were introduced into co-cultures of BALB/c splenocytes and C57BL/6 UmAdMSCs, FucmAdMSCs or sialFucmAdMSCs (MSC:splenocyte ratio of 1:20) previously adhered to HA or mE-Ig for 72 h. Mitogen-induced splenocyte proliferation was calculated by subtracting the level of splenocyte basal proliferation in the absence of ConA. Addition of SB-431542 (TGFβ inhibitor), 1-methyl-DL-tryptophan (1-MT) (IDO inhibitor), or N^G^-monomethyl-L-arginine (L-NMMA) (iNOS inhibitor) led to significant increases in proliferation of responder splenocytes (**p < 0.01 or ***p < 0.001). Splenocyte proliferation was significantly decreased compared to same conditions in absence of HA or E-selectin, ^δδ^*p* < 0.01, or increased compared to same conditions using FucmAdMSCs, ^##^*p* < 0.01, respectively. All data are presented as the mean ± SD of *n* = 3 separate experiments and analyzed by one-way ANOVA with Tukey’s multiple-comparisons test.

To ascertain whether MSC-lymphocyte cell–cell contacts are mandatory for the observed anti-proliferative effects of MSC CD44/HCELL ligation, we obtained supernatants of HCELL^−^ mAdMSCs and HCELL^+^ mAdMSCs after engagement with either HA or E-selectin and tested the effects of conditioned media on mitogen-induced splenocyte proliferation. As shown in Fig. 5g, supernatants obtained from HA-ligated UmAdMSCs or HA-ligated sialFucmAdMSCs, and also from HA-ligated or E-selectin-ligated FucmAdMSCs, in each case profoundly inhibited mitogen-induced splenocyte proliferation (Fig. 5g, right) to an extent similar to that observed in continuous presence of MSCs (Fig. 5g, left). These findings indicate that secreted products of MSCs drive the observed dampening of splenocyte proliferation. Accordingly, to elucidate the relevant molecular effector(s), we analyzed the supernatant levels of TGFβ, IDO, nitrates/nitrites (e.g., nitric oxide (NO) metabolites), PGE_2_, and IL-10, each of which are reported to mediate immunosuppression. As shown in Fig. 6a–c and Supplementary Fig. 2, engagement of HCELL via E-selectin and of CD44 via HA on mAdMSCs, in each case, profoundly boosts levels of TGFβ, IDO, and NO metabolites (Fig. 6a–c); however, levels of IL-10 and PGE_2_ on murine AdMSCs are unaffected by CD44/HCELL ligation (Supplementary Fig. 2a, b). To further analyze the contributions of TGFβ, IDO, and NO to the observed mAdMSC anti-proliferative effect, we performed in vitro mMSC:splenocyte co-cultures in presence of inhibitors of these molecules at mAdMSC:splenocyte ratio of 1:20 (Fig. 6d) and of 1:10 (Supplementary Fig. 3), with or without pre-incubation with HA or E-selectin. Addition of SB-431542 (TGFβ inhibitor), 1-methyl-DL-tryptophan (IDO inhibitor), or N^G^-monomethyl-L-arginine (iNOS inhibitor) in each case significantly rescued the proliferation of mitogen-stimulated splenocytes, and simultaneous use of all three inhibitors resulted in complete recovery of proliferation (Fig. 6d). Collectively, these results indicate that MSC-secreted molecules license mAdMSC immunomodulatory effects on activated splenocytes.

**Fig. 7:**
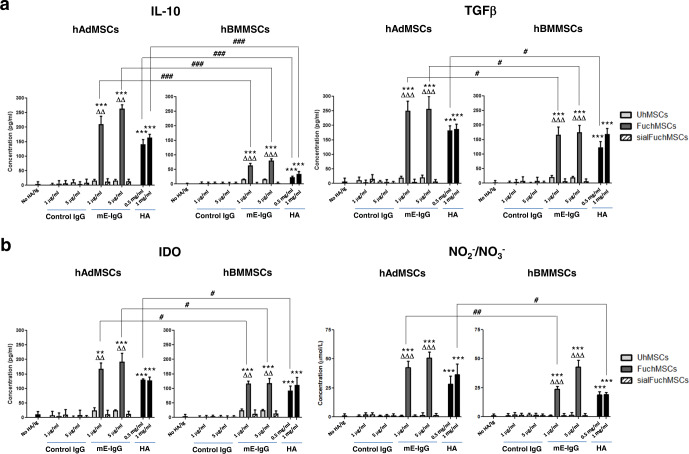
Effects of HCELL/CD44 engagement on immunomodulatory properties of human MSCs. Human MSCs derived from adipose tissue (hAdMSCs) or bone marrow (hBMMSCs) were fucosylated (“Fuc”: FuchAdMSCs or FuchBMMSCs) or buffer-treated (unmodified (“U”): UhAdMSCs or UhBMMSCs) and cultured in presence of different concentrations of E-selectin (mE-Ig) or hyaluronic acid (HA) for 3 days at 37 °C. Thereafter, culture supernatants were harvested and analyzed by ELISA for **a** levels of anti-inflammatory molecules interleukin-10 (IL-10) and TGFβ, or **b** IDO and NO metabolites (e.g., NO^2−^/NO^3−^). Cells cultured in the absence of HA or in presence of control IgG served as negative controls. Also, as controls to assess specificity of E-selectin binding, FuchAdMSCs or FuchBMMSCs were treated with sialidase (“sialFuchAdMSCs” or “sialFuchBMMSCs”) to cleave terminal sialic acid from sLe^x^ (thereby abrogating binding to E-selectin). As shown in panels, levels of analyzed immunomodulatory molecules significantly increased following hMSC co-incubation with either E-selectin or HA (**p < 0.01 or ***p < 0.001); levels of immunomodulatory molecules did not rise in sialFuchMSCs co-incubated with E-selectin (compared to FuchMSCs, ^ΔΔ^*p* < 0.01 or ^ΔΔΔ^*p* < 0.001, respectively). Overarching bar lines reflect comparisons of immunomodulatory molecule levels between supernatants of hAdMSCs and of hBMMSCs following co-incubation with HA or E-selectin: notably, supernatant levels of all analyzed immunomodulatory molecules were significantly higher (differences of ^#^*p* < 0.05, ^##^*p* < 0.01, or ^###^*p* < 0.001, as shown) in cultures of adipose-derived hMSCs compared to those of bone marrow-derived hMSCs, most conspicuously for IL-10 (^###^*p* < 0.001). Data are presented as the mean ± SD of *n* = 3 separate experiments and analyzed by one-way ANOVA with Tukey’s multiple-comparisons test.

To analyze whether the observed immunomodulatory effect of CD44/HCELL engagement in murine MSCs is also characteristic of human MSCs (hMSCs), we measured the levels of secreted anti-inflammatory molecules after CD44/HCELL ligation of HCELL^+^ (i.e., exofucosylated) and HCELL^−^ hMSCs derived from both adipose tissue (hAdMSCs) and bone marrow (hBMMSCs) sources. Strikingly, just as observed in murine MSCs, both hAdMSCs and hBMMSCs each produced markedly higher levels of TGFβ, of IDO, and of NO metabolites after HCELL or CD44 ligation to E-selectin or HA, respectively (Fig. 7a, b). However, in stark contrast to murine MSCs, CD44/HCELL ligation also profoundly boosted the production of the anti-inflammatory cytokine IL-10 by human MSCs from both marrow and adipose tissue sources (Fig. 7a). Notably, under stimulation by CD44/HCELL ligation with HA or E-selectin, respectively, production of all analyzed immunomodulatory molecules was much higher in hAdMSCs than in hBMMSCs, especially for IL-10 (Fig. 7a, b and Table 1). Indeed, among hAdMSCs, when analyzed as a ratio between IL-10 levels in hMSCs that engaged/did not engage either E-selectin or HA (i.e., ratio of FuchAdMSCs/UhAdMSCs for E-selectin exposure or of UhAdMSCs with HA exposure/UhAdMSCs without HA exposure), IL-10 production was heightened 10-fold by engagement of either HCELL (via E-selectin) or of CD44 (via HA); furthermore, following CD44/HCELL ligation, adipose-derived hMSCs consistently showed >3-fold higher production of IL-10 compared to that of bone marrow-derived hMSCs (Table 1).

**Table 1:** Comparisons of the levels of soluble immunomodulatory molecules in culture supernatants after E-selectin-mediated HCELL ligation (ratios of Fucosylated (“Fuc”)/unmodified (“U”) hMSCs) or HA-mediated CD44 ligation (ratios of HA-exposed (“w/HA”)/unexposed (“w/o HA”)) among culture-expanded adipose-derived human MSCs (hAdMSCs) and bone marrow-derived human MSCs (hBMMSCs).

Anti- inflammatory molecule	Ratio FuchAdMSCs/UhAdMSCs	Ratio FuchBMMSCs/UhBMMSCs	Ratio FuchAdMSCs/UhAdMSCs	Ratio FuchBMMSCs/UhBMMSCs	Ratio UhAdMSCs w/HA/UhAdMSCs	Ratio UhBMMSCs w/HA/UhBMMSCs	Ratio UhAdMSCs w/HA/UhAdMSCs	Ratio UhBMMSCs w/HA/UhBMMSCs
	mE-Ig (1 μg/ml)	mE-Ig (1 μg/ml)	mE-Ig (5 μg/ml)	mE-Ig (5 μg/ml)	w/o HA HA (0.5 mg/ml)	w/o HA HA (0.5 mg/ml)	w/o HA HA (1 mg/ml)	w/o HA HA (1 mg/ml)
IL-10	12.74 ± 1.64***	3.87 ± 0.41	15.96 ± 0.81***	4.85 ± 0.41	9.58 ± 0.96***	2.42 ± 0.23	10.96 ± 0.67***	3.13 ± 0.47
TGFβ	12.16 ± 1.58*	8.11 ± 1.27	12.42 ± 2.10*	8.50 ± 1.13	9.84 ± 0.79*	6.99 ± 0.95	10.10 ± 0.81	9.20 ± 0.93
IDO	6.75 ± 0.80*	4.71 ± 0.33	7.72 ± 1.18*	4.74 ± 0.68	6.23 ± 0.14*	4.73 ± 0.63	6.16 ± 0.46	5.50 ± 1.05
NO_2_^−^/NO_3_^−^	5.12 ± 0.52**	3.31 ± 0.24	5.90 ± 0.51	5.14 ± 0.58	3.73 ± 0.68	2.82 ± 0.27	4.52 ± 0.89*	2.86 ± 0.17

*p < 0.05, **p < 0.01, ***p < 0.001, respectively, according to one-way ANOVA with Tukey’s multiple-comparisons test.

To further analyze the functional impact of the engagement of CD44/HCELL to HA/E-selectin, respectively, we investigated whether ligation of CD44/HCELL affects mAdMSC adhesion to fibronectin, one of the critical components of the extracellular matrix whose expression is markedly upregulated by inflammation^32^. Cellular adhesion to fibronectin is mediated by β1 integrins, principally integrins VLA-4 and VLA-5, both of which are characteristically expressed on all MSCs. As shown in Supplementary Fig. 4, mAdMSCs were exposed to HA or E-selectin and subsequently analyzed for fibronectin binding. FucmAdMSCs previously exposed to E-selectin, or UmAdMSCs and FucmAdMSCs previously exposed to HA, significantly upregulate β1 integrin-mediated adhesion to mouse fibronectin (mFN), whereas treatment of FucmAdMSCs with sialidase (sialFucmAdMSCs), or treatment of the mAdMSCs with β1-function-blocking HMβ1-1 antibody, significantly dampens this effect. Thus, beyond inducing release of immunomodulatory agents, CD44/HCELL ligation activates VLA-4/VLA-5-mediated adhesion to fibronectin, boosting cellular colonization within sites of inflammation.

## Discussion

When triggered, the immune system must engage with sufficient potency to vigorously defend against the inciting agent/pathogen, but, concomitantly, must also be subject to constraint(s) to prevent destroying the host itself. Despite decades of studies on the pathobiology of immune disorders, we still have limited knowledge into how native components of the tissue microenvironment function to ameliorate or prevent immunopathology in a site-specific manner. In “systemic” immune diseases, the presence of focal tissue-sparing within affected target organ(s) reflects the presence/persistence of immunohomeostasis at those pertinent unaffected site(s), i.e., the preservation of native tissue integrity in the face of overt inflammation in anatomically adjacent segments indicates that sufficient amounts/density of immunomodulatory cellular elements are microenvironmentally present in situ to restrain the action(s) of inflammatory effectors. Ideally, these effectors of immunoquiescence would be broadly distributed within all tissues, prompted into action as warranted by the local inflammatory milieu.

One potential cellular effector of immunohomeostasis is the MSC. Every tissue of the body contains a reservoir of MSCs, but MSC distribution(s) in situ cannot be analyzed/characterized because there are no surface markers that uniquely identify these cells. Nonetheless, MSCs can be harnessed from tissues and subsequently culture-expanded for therapeutic purposes. However, upon intravascular administration, these culture-expanded MSCs are largely trapped in the afferent vessels of the lungs and rapidly cleared^33,34^, prompting the need to create strategies to optimize their accumulation at affected tissues. Another hurdle to realizing their full potential in treatment of immunopathologic processes is that vascularly-administered MSCs cannot effectively colonize inflammatory sites because these cells natively lack effectors of cell migration such as E-selectin ligands that can guide their extravasation at affected endothelial beds^17^. Thus, in order to program lesional intercalation of mAdMSCs at the onset/progression of immune-mediated tissue injury, we enforced expression of the potent E-selectin ligand HCELL on mAdMSCs prior to systemic administration of these cells. Because donor splenocyte-enriched full MHC-mismatched transplant in mice reproducibly triggers aGvHD, a fulminant immunopathologic process, we reasoned that steering the migration of MSCs to the anatomic sites of incipient aGvHD tissue injury would reveal whether MSC tissue colonization can promote and/or preserve immunohomeostasis.

Previous studies in a murine model of aGvHD reported that mice receiving early infusion (at days 0, +7, and +14 post-transplant) of mAdMSCs showed less aGvHD than those receiving cells at days +14, +21, and +28 post-transplant^35^. Following intravenous administration of recipient-type HCELL^+^ GFP^+^ or HCELL^−^ GFP^+^ mAdMSCs in animals with aGvHD in the early post-transplant period, immunohistochemical studies consistently revealed increased recruitment of MSCs into aGvHD-affected sites in animals receiving HCELL^+^ AdMSCs compared to those receiving HCELL^−^ AdMSCs. The increased tissue residency of HCELL^+^ AdMSCs was associated with significant blunting of the severity of the evolving aGvHD, with strikingly increased animal survival compared to those receiving HCELL^−^ AdMSCs (and to those that did not receive MSCs (“Untreated”)). Notably, compared to early post-transplant administration of HCELL^−^ AdMSCs, administration of HCELL^+^ AdMSCs yielded increased MSC residency within the gut and liver parenchyma, and dramatically increased MSC tropism to intestinal lamina propria. We did not detect MSC infiltrates in lymphoid tissues of mice receiving either type of AdMSCs, indicating that MSC infiltration within aGvHD-affected tissue itself, and not enhanced colonization of lymphoid tissues, elicits the observed immunomodulatory effect. Though mice that received HCELL^+^ AdMSCs were not completely devoid of disease, a much lower lesional spectrum was observed: histological analysis revealed that administration of HCELL^+^ AdMSCs markedly attenuated aGvHD damage, prominently within the gastrointestinal tract and liver, compared to that observed in mice receiving HCELL^−^ AdMSCs and in Untreated mice. Moreover, the administration of HCELL^+^ AdMSCs yielded durable suppression of aGvHD, whereas administration of HCELL^−^ AdMSCs engendered only a partial and transient capacity to prevent/reverse aGvHD.

The immunopathology of aGvHD is driven by immunoreactive donor effector T cells within target tissues, a process that takes place even during periods of lymphopenia and before engraftment^23^. As expected, analysis of T cell infiltrates within liver and gut of animals with ongoing aGvHD showed prominent presence of these cells early post-transplant. Compared to levels of T cell infiltrates in untreated mice, administration of HCELL^−^ AdMSCs resulted in a modest decrease in T cell infiltrates in these organs. However, in mice receiving HCELL^+^ AdMSCs, comparatively lower T cell infiltrates were consistently observed at 10 and 20 days post-transplant, with substantially reduced degree of tissue damage. Notably, the pattern of neutrophil infiltration mirrored that of T cells: following neutrophil engraftment (i.e., beyond day +10), markedly lower gut and liver infiltrates were observed in those mice receiving HCELL^+^ AdMSCs. Lower levels of neutrophil infiltrates within aGvHD-target organs correlate with better prognosis^28^, as these cells contribute to tissue damage both directly (e.g., by release of ROS) and indirectly through the promotion of T cell activation^36,37^.

The infiltration of effector immune cells into tissues is associated with release of pro-inflammatory cytokines that have harmful effects in the affected organs and play a crucial role in the pathophysiology of immune-mediated diseases such as aGvHD^38,39^. We observed here that mice treated with mAdMSC showed significantly decreased plasma levels of several pro-inflammatory cytokines compared to that of Untreated mice, yet this effect was not sustained in mice receiving HCELL^−^ AdMSCs (i.e., pro-inflammatory cytokine levels in UmAdMSC-treated mice dropped then gradually increased to that observed in Untreated animals by day +30) (Fig. 4). In contrast, animals treated with HCELL^+^ AdMSCs displayed a marked and prolonged decrease in plasma levels of pro-inflammatory mediators, and these mice had significant increases in plasma levels of anti-inflammatory cytokines IL-10 and TGFβ (Fig. 4), each of which potently inhibit lymphocyte proliferation and promote tolerance^40,41^. In particular, increased levels of IL-10 could in itself profoundly dampen immunoreactivity, as this cytokine, initially recognized as the “cytokine synthesis inhibitory factor” (CSIF)^42–46^, has pleiotropic effects in not only decreasing the production of a variety of pro-inflammatory cytokines, but also in inhibiting T cell proliferation and decreasing the expression of MHC molecules and costimulatory molecules on antigen-presenting cells^47,48^; in fact, through its potent role in immunosuppression, IL-10 is known to mediate tissue preservation in the face of exaggerated host immune responses to pathogens, thereby establishing host-pathogen immune equilibrium resulting in infectious latency^49^. The role(s) of IL-10 in mediating immunomodulation following the administration of HCELL^+^ MSCs merits additional investigation. Moreover, apart from the observed increased plasma levels of anti-inflammatory cytokines, the ability of MSC administration to diminish pro-inflammatory cytokine levels would also serve to promote tissue preservation, limiting the systemic extension of immunopathology by decreasing immunoresponsiveness and decreasing the (cytokine-driven) induction of endothelial adhesion molecules that mediate recruitment of immune effector cells to inflammatory sites^50^. Further studies are warranted to evaluate the extent to which the observed salutary effects of systemically administered HCELL^+^ MSCs are consequent to the dampening of levels of pro-inflammatory agents versus the augmentation of plasma levels of anti-inflammatory agents, and the relative impact of fluxes in particular agents versus combinations thereof.

Our results indicate that MSC-lymphocyte cell–cell contacts are not mandatory for MSC attenuation of mitogen-induced lymphocyte proliferation. Indeed, supernatants obtained from HCELL^+^ AdMSCs and from HCELL^−^ AdMSCs after pre-incubation with either E-selectin or HA, respectively, equally reduced mitogen-induced splenocyte proliferation to levels observed with continuous MSC contact (Fig. 5). These results indicate that secreted products of MSCs drive the observed anti-proliferative property. Analysis of the expression of TGFβ, IL-10, IDO, nitric oxide (NO) metabolites, and PGE_2_ indicate that in vitro engagement of HCELL via E-selectin or HA, or of CD44 via HA, in each case profoundly boosts levels of TGFβ, IDO, and NO metabolites in both mouse (Fig. 6) and human MSCs (Fig. 7). Interestingly, ligation of HCELL or of CD44 in murine MSCs did not directly increase production of IL-10 (Supplementary Fig. 2). These data suggest that the observed increased plasma levels of IL-10 in mice receiving MSCs may be secondary to well-recognized indirect effects of MSCs in supporting/upregulating IL-10 production among other cell types that express this cytokine (e.g., monocytes, macrophages, dendritic cells, B cells and subsets of T cells)^47^, and this function of MSCs has been reported to be potent enough in itself to drive immunomodulation in a variety of contexts^51–53^. In contrast, ligation of HCELL or of CD44 among human MSCs directly and strikingly boosts production of IL-10 (as well as TGFβ, IDO, and nitrates/nitrites) (Fig. 7). These findings are consistent with prior studies reporting species-specific variations in MSC immunomodulatory mechanisms (such as increased production of IDO by human MSCs or a sustained expression of iNOS by mouse MSCs^54^). Moreover, it is known that MSCs derived from different tissue sources within the same mammal have variations in immunomodulatory properties. Herein, our data indicate that following HCELL or CD44 engagement, adipose-derived human MSCs have much higher production of these agents than do bone marrow-derived human MSCs, and, conspicuously, IL-10 production by adipose-derived human MSCs is most profoundly induced (Fig. 7 and Table 1). Direct production of IL-10 by resting (i.e., not cytokine-treated) human MSCs (or by mouse MSCs) has not been reported previously^55^, and the observed marked increased production of anti-inflammatory cytokines, especially IL-10, by human adipose-derived MSCs compared to human bone marrow-derived MSCs could be a factor underlying the higher immunomodulatory activity of these cells compared to that of bone marrow-derived human MSCs as reported in some studies^56,57^. Interestingly, the observed robust production of IL-10 by human adipose-derived MSCs may also reflect non-immunologic functions of this cytokine within adipose tissue as emerging data indicate that IL-10 could promote “metabolic syndrome” by its effects on limiting energy utilization and thermogenesis by adipocytes^58^.

The heightened production of TGFβ, IDO, and NO following engagement of FucmAdMSCs with E-selectin in vitro indicates that, following engagement with E-selectin displayed on vascular beds at inflammatory sites in vivo, HCELL^+^ mAdMSCs are primed to exert immunoquiescent effects within the inflammatory milieu via increased release of anti-inflammatory molecules^59–62^. This mechanism, together with the observed higher MSC tissue density in situ, underlies the observed improved clinical outcome of mice with fulminant immunoreactivity. Moreover, independent of E-selectin/HCELL interactions, engagement of MSC CD44 with its cognate ligand, HA, similarly boosts TGFβ, IDO, and NO production. Thus, once extravasated and localized within tissue parenchyma, MSC immunomodulatory properties would be unleashed through interaction of CD44 with HA, an integral component of the extracellular matrix whose expression is itself upregulated at sites of immunoreactivity/inflammation^63,64^, and has itself been linked to immunoregulatory effects^65^. Thus, the results of this study offer novel mechanistic perspectives on how molecules within the inflammatory milieu can boost MSC capabilities as effectors of immunohomeostasis. Moreover, the data suggest that engagement of MSC CD44/HCELL with ligands such as HA/E-selectin prior to infusion of the cells could be exploited to potentiate the capacity of administered MSCs to dampen immunopathology in vivo. Future experiments in our laboratory will focus on unraveling the specific downstream signaling cascades triggered by CD44/HCELL ligation. Importantly, we observed that engagement of HCELL^+^ mAdMSCs with E-selectin, or mAdMSC CD44/HCELL engagement with HA, in each case triggers β1 integrin-mediated adhesiveness, resulting in enhanced mAdMSC binding to fibronectin in the absence of exogenous chemokine stimulation^21^. Therefore, upon entering the tissue parenchyma, previous HCELL binding to E-selectin or HCELL/CD44 engagement to HA could boost lodgment of mAdMSCs within the pertinent inflammatory microenvironment(s).

Current therapies for pathologic immunoreactivity, and for immune-mediated diseases in general, are primarily pharmacologic. New therapeutic approaches are urgently needed to improve patient outcomes as current pharmacologic agents produce broad-spectrum immunosuppression and are associated with significant adverse effects. Ideally, therapeutic immunomodulation should be concentrated solely at the site(s) of immunopathology, thereby establishing anatomically focal immunohomeostasis and preserving systemic immunoprotection. Our findings indicate that MSC organ/tissue intercalation engenders refractoriness to the pathologic consequences of immune-mediated inflammatory processes in situ. Accordingly, inflammation-induced expression of E-selectin within endothelial beds at affected sites could be leveraged for clinical benefit to achieve efficient tissue residency of systemically administered E-selectin ligand-bearing immunomodulatory MSCs at the desired anatomic location(s). Thus, rather than antagonizing E-selectin bioactivity (e.g., by use of anti-E-selectin mAb or mimetics of sLe^X^) or its expression (e.g., by use of biologics blocking TNF or IL-1 action)^66^, pathophysiologic endothelial E-selectin display could serve as a gateway in ushering forth a new era of immunoregulatory cell-based therapies for inflammatory disorders.

## Methods

### Mice

BALB/c (H-2^d^) donors and C57BL/6J (H-2^b^) recipient mice were purchased from Envigo, whereas β-actin-GFP transgenic C57BL/6-Tg (CAG-EGFP) was from The Jackson Laboratory. All animal procedures were approved by the Institutional Animal Care and Use Committee at University of Murcia (Murcia, Spain) and performed according to the guidelines of our Institution (approved protocol A13150201).

### Mesenchymal stem cell isolation and culture

Murine AdMSCs (mAdMSCs), human AdMSCs (hAdMSCs), and human BMMSCs (hBMMSCs) were isolated from mouse epididymal fat pads, and human lipoaspirates and bone marrow harvests, respectively. In brief, mAdMSCs from C57BL/6 or C57BL/6-Tg (CAG-EGFP) mice, or hAdMSCs and hBMMSCs from healthy human donors (*n* = 3 for each source), were flask-seeded in DMEM low glucose medium (Gibco) supplemented with 15% fetal bovine serum (Gibco), 1% L-glutamine (Lonza), 100 U/ml penicillin, and 100 μg/ml streptomycin (Lonza) (complete medium). MSCs in culture passages 3–4 were used for experiments. The Institutional Review Board of the University Hospital Virgen de la Arrixaca (Murcia, Spain) approved the protocols used to obtain and process all human samples. As needed, written informed consent was obtained from donors as per Helsinki Declaration guidelines.

### Murine model of aGvHD

Fully MHC-mismatched allo-HSCT was performed by transplanting bone marrow cells from donor BALB/c mice into 10-week-old C57BL/6 recipients previously irradiated with a potentially lethal dose of 10 Gy divided into two doses of 5 Gy spaced 24 h apart (days −1 and +0). On day +0 recipient mice were transplanted intravenously with 1 × 10^7^ bone marrow cells from donor mice, either without (i.e., whereby aGvHD did not develop) or with 1.5 × 10^7^ donor splenocytes to induce aGvHD. Those mice treated with mAdMSC received intravenous infusions of 5 × 10^4^ recipient-type UmAdMSC or FucmAdMSCs on days 0, +7, and +14 post-transplantation. Survival of animals after transplantation was monitored daily whereas clinical aGvHD was assessed using a scoring system that generates a composite aGvHD score composed of individual scores for weight loss, posture, activity, skin integrity and fur texture.

### Fucosyltransferase VII treatment

Murine AdMSCs were derived from C57BL/6 mice, the recipient strain for MHC-mismatched HSCT, whereas hAdMSCs and hBMMSCs were isolated from healthy human donors. Fucose was stereoselectively installed onto sialyllactosaminyl glycans of CD44 using an α(1,3)-linkage-specific fucosyltransferase, fucosyltransferase VII (FTVII; obtained from R&D Systems), in presence of donor fucose substrate (GDP-fucose; Sigma Aldrich): MSCs were resuspended at 2 × 10^7^ cells/ml and incubated for 60 min at 37 °C in FTVII reaction buffer composed of Hank’s Balanced Salt Solution (HBSS) (without Ca^2+^ and Mg^2+^) (Lonza) containing 20 mM HEPES (Lonza), 0.1% human serum albumin (HSA) (Grifols), 30 μg/ml FTVII (R&D Systems), and 1 mM GDP-fucose (Fucosylation-modified, “FucmAdMSCs”). Controls consisted of MSCs treated with reaction buffer alone (i.e., Unmodified MSCs, “UmAdMSCs” or, “UhAdMSCs” and “UhBMMSCs”). Exofucosylation efficacy was measured by analysis of HECA452 antibody (10 μg/ml, BD Biosciences, Cat#555946) staining and murine E-selectin-human Fc chimera (mE-Ig; 5 μg/ml, R&D Systems, Cat#575-ES-100) binding by flow cytometry and western blot.

### Mitogen proliferative assays

For mitogen proliferative assays, splenocytes were isolated from C57BL/6 and BALB/c mice spleen cell suspensions by passage through 40-μm nylon cell strainers (Becton Dickinson), followed by red blood cell lysis buffer containing 0.83% ammonium chloride in 0.01 M Tris-HCl buffer pH 7.5 (Sigma Aldrich). To induce splenocyte proliferation, 1 × 10^5^ splenocytes were resuspended in RPMI 1640 medium (Sigma Aldrich) supplemented with 10% FBS (proliferation medium) and treated with 10 μg/ml concanavalin A (ConA) (Sigma Aldrich). To assess for effect(s) on splenocyte proliferation, MSCs were resuspended in complete medium and seeded in wells together with splenocytes at decreasing ratios of MSC:splenocyte (from 1:1 to 1:100). After 3 days of MSC:splenocyte co-cultures, splenocyte proliferation was measured using an ELISA BrdU colorimetric kit (Roche Diagnostics). In brief, BrdU labeling reagent was added to wells 16 h before determination. Then, cells were fixed, DNA denatured, and incubated with an anti-BrdU-POD antibody. After washing and substrate addition, absorbance was measured. In some experiments, UmAdMSCs and FucmAdMSCs, or FuchAdMSCs and FuchBMMSCs, were treated with sialidase from *Vibrio cholerae* (0.1 U/ml, Roche Diagnostics) to remove terminal sialic acids (i.e., sialUmAdMSCs and sialFucmAdMSCs, or sialFuchAdMSCs and sialFuchBMMSCs). As indicated, mMSCs or hMSCs were cultured for 24 h or 72 h with different concentrations of mE-Ig chimera or of hyaluronic acid (HA, from rooster comb; Sigma Aldrich) for 24 h or 72 h. Briefly, mE-Ig or HA were immobilized on plates. HA-coated plates were incubated with 3% BSA in DMEM medium to block non-specific interactions. Thereafter, mAdMSCs or hMSCs were cultured in cell adhesion media consisting of HBSS medium containing 2 mM CaCl_2_, 10 mM HEPES, 0.2% BSA, and 1 mM sodium pyruvate (for mE-Ig), or DMEM medium containing 10 mM HEPES, 0.2% BSA and 1 mM sodium pyruvate (for HA), respectively. To block CD44/HCELL interactions to HA, a purified rat anti-mouse CD44 antibody (clone KM114, 10 μg/ml, Santa Cruz Biotechnology, Cat#sc-18882) were employed. In some experiments, cultures of mAdMSCs seeded on E-selectin and/or HA for 72 h were washed, then co-cultured in presence of continuous E-selectin and/or HA with murine splenocytes (at MSC:splenocyte ratio of 1:20) for 72 h in presence of ConA, and splenocyte proliferation was assessed. To examine the contribution of different anti-inflammatory molecules in the immunosuppressive properties of mAdMSCs, the TGFβ inhibitor SB-431542 (final concentration (Cf) = 10 μM), the IDO inhibitor 1-methyl-DL-tryptophan (Cf = 1 μM) or the iNOS inhibitor N^G^-monomethyl-L-arginine (Cf = 1 mM) (all from Sigma Aldrich) was added at the beginning of co-culture with splenocytes. To analyze the effects of HCELL/CD44 ligation by either E-selectin/HA in immunoregulatory molecules production, hAdMSCs and hBMMSCs were adhered to E-selectin or HA for 72 h, washed, co-cultured at MSC:T cell ratio of 1:20 for 72 h with human peripheral blood T cells in presence of phytohemagglutinin (PHA, Sigma Aldrich) and supernatants recollected for immunomodulatory molecules analysis. Finally, to evaluate the β1 integrin-dependent adhesion of mAdMSCs after CD44/HCELL ligation, 96-well plates were coated with 10 μg/ml of mouse fibronectin (mFN) (Abbexa), incubated overnight at 4 °C and blocked with 3% BSA in PBS for 2 h at 37 °C. After, UmAdMSCs, FucmAdMSCs, or sialFucmAdMSCs, previously exposed to E-selectin (5 μg/ml) or to HA (1 mg/ml) for 1 h at 37 °C, were detached with 5 mM EDTA in PBS, labeled with 2,7-bis(carboxyethyl)-5(6)-carboxyfluorescein-acetoxymethyl ester (BCECF-AM; Sigma Aldrich), added to mFN-coated wells and allowed to adhere for 1 h at 37 °C. To assess specificity and function of β1 integrin-mediated adhesion to mFN, some UmAdMSCs or FucmAdMSCs were previously incubated with blocking anti-β1 antibody (clone HMβ1-1, 10 μg/ml, BioLegend, Cat#102201) for 20 min at 4 °C, or exposed to 1 mM MnCl_2_ for 1 min at R/T, respectively. Finally, non-bound cells were removed by washing with PBS and adhered cells were lysed with 0.1% SDS in PBS. Extent of adhesion was then quantified using a fluorescence microplate reader (Tecan).

### Histopathology analysis

Histopathological changes of aGvHD were analyzed in at least two distant areas of liver, gut (colon), and skin by a single pathologist blinded to the treatment groups. Samples from all organs were collected and fixed in 4% neutral buffered formaldehyde for 24 h, processed and paraffin-embedded. Three-μm-thick sections were then obtained and stained with a standard hematoxylin and eosin (H&E) staining for routine histopathological analysis. The skin histopathologic lesions were graded as follows: grade 0 (normal), grade I (slight vacuolar degeneration of epidermal basal cells), grade II (scattered individual apoptotic epidermal basal cells and spongiosis), grade III (separation of dermo-epidermal junction) and grade IV (diffuse and severe ulceration, extensive destruction of epidermis). The scoring system for gut was: grade 0 (normal), grade I (scattered individual apoptotic cells and inflammatory cell infiltrate), grade II (crypt epithelial cell apoptosis, villous blunting, exploding crypts), grade III (focal mucosal ulceration and moderate villous atrophy) and grade IV (diffuse and severe mucosal ulceration). Histopathologic changes of liver sections were scored as: grade 0 (normal), grade I (epithelial damage and ≤25% bile ducts affected), grade II (epithelial damage and 25–49% bile ducts affected), grade III (epithelial damage and 50–74% bile ducts affected), and grade IV (epithelial damage and ≥75% bile ducts affected). In addition, polymorphonuclear neutrophils (PMNs) were identified on H&E stained sections on basis to its morphological features (segmented nuclei). A standard indirect ABC immunohistochemical staining was performed in sections from all organs. Briefly, after deparaffination, rehydration, antigen demasking, and peroxidase-blocking, sections were incubated with a polyclonal rabbit anti-CD3 antibody (1:500, Agilent Technologies, Cat# A0452) for 1 h at 37 °C. In other experiments and to detect the distribution of transplanted GFP-expressing mAdMSCs, sections were incubated with a polyclonal chicken anti-GFP antibody (1:4000, Aves Labs, Cat#GFP-1020). Analysis of endothelial E-selectin (1:100, Abcam, Cat#ab18981) and CD31 (1:100, Abcam, Cat#ab28364) co-localization was performed on sequential sections. After washing, sections were incubated with a secondary anti-rabbit labeled polymer (EnVision®, Agilent Dako) for 20 min at 37 °C. Finally, immunolabeling was revealed using 3-3′-diaminobencidine (DAB) and counterstained with hematoxylin. Positive reaction was identified as a dark-brown precipitated with a membrane or cytoplasmic pattern for CD3, E-selectin, and CD31 or GFP staining, respectively. Histopathologic and immunohistochemical analysis was performed using a standard light microscope (Zeiss Axio A10, Carl Zeiss).

### Quantification of cytokines and nitric oxide

Murine IFN-γ, IL-1β, TNF-α, TGFβ, IL-10, IL-12, IL-6, IL-17, PGE_2_, and IDO were quantified in plasma of animals or culture supernatants by ELISA (RayBiotech, Diaclone, bioNova Cientifica, Elabscience, and Cusabio Biotech). Human TGFβ, IDO, and IL-10 ELISA kits were purchased from RayBiotech and Elabscience. Nitric oxide was detected in culture supernatants using a modified Griess reagent (Parameter™ total nitric oxide and nitrate/nitrite assay, R&D Systems). Briefly, all nitrates are converted into nitrites by nitrate reductase, and total nitrites detected by the Griess reaction. Samples and standards were analyzed in triplicates according to the manufacturer’s instructions.

### Statistics

Data are expressed as mean ± SD. The number of independent experimental replicates is indicated in figure legends, with *n* representing the number of replicates for in vitro experiments or number of animals used per experimental group. Comparisons between groups were analyzed using one-way ANOVA followed by Tukey’s post hoc comparisons test. Survival curves were plotted using Kaplan–Meier estimates and statistically analyzed using the Mantel–Cox log-rank test. Correlation was determined by using the Pearson correlation coefficient. *P* values <0.05 were considered statistically significant. GraphPad prism 5.0 was used to perform statistical analyses and to generate graphs.

### Reporting summary

Further information on research design is available in the Nature Research Reporting Summary linked to this article.

## Supplementary information

Supplementary Information

REPORTING SUMMARY

## Data Availability

The data that support the findings of this study are available from the corresponding author upon request.
