# Supplementary material for: Enforced mesenchymal stem cell tissue colonization counteracts immunopathology
Source: NPJ Regen Med. 2022 Oct 19;7:61. doi: 10.1038/s41536-022-00258-z (PMC9582223; doi:10.1038/s41536-022-00258-z)
Supplement: Supplementary file 1 — Supplementary Information [file 41536_2022_258_MOESM1_ESM.pdf]

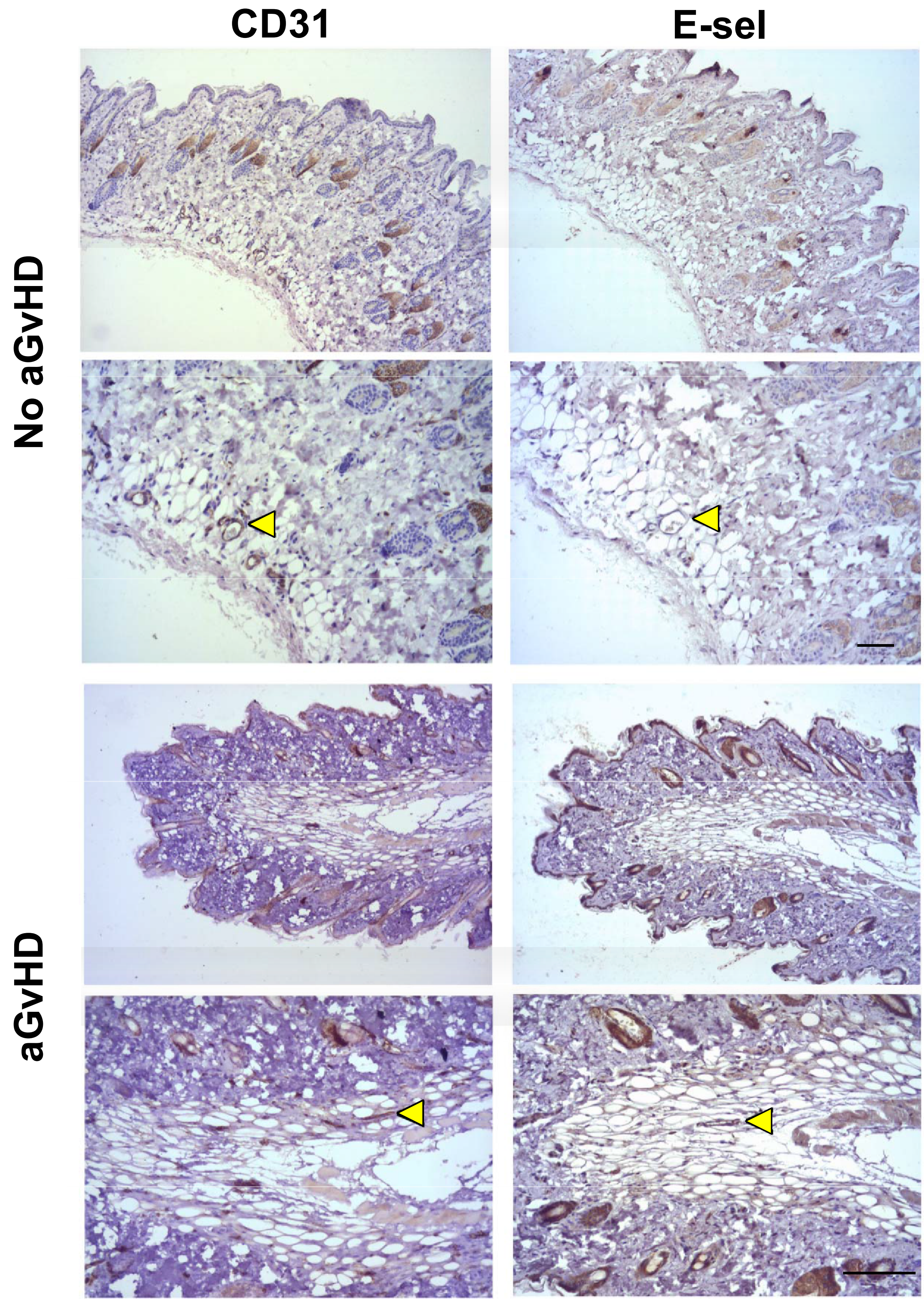

Supplementary Fig. 1 E-selectin expression is upregulated in skin microvessels in aGVHD. Staining of sequential sections of C57BL/6 skin showing colocalization of E-selectin and endothelial marker CD31 (delimited by arrowheads) in mice with aGvHD (bottom images) and in those mice without aGvHD (top images, "no aGvHD"; *i.e.,* mice that received allogeneic marrow alone). Top images x100 magnification; bottom images x200 magnification, Scale bar: 100 μm. Images are representative of n=3 separate experiments, n=8 animals per group.

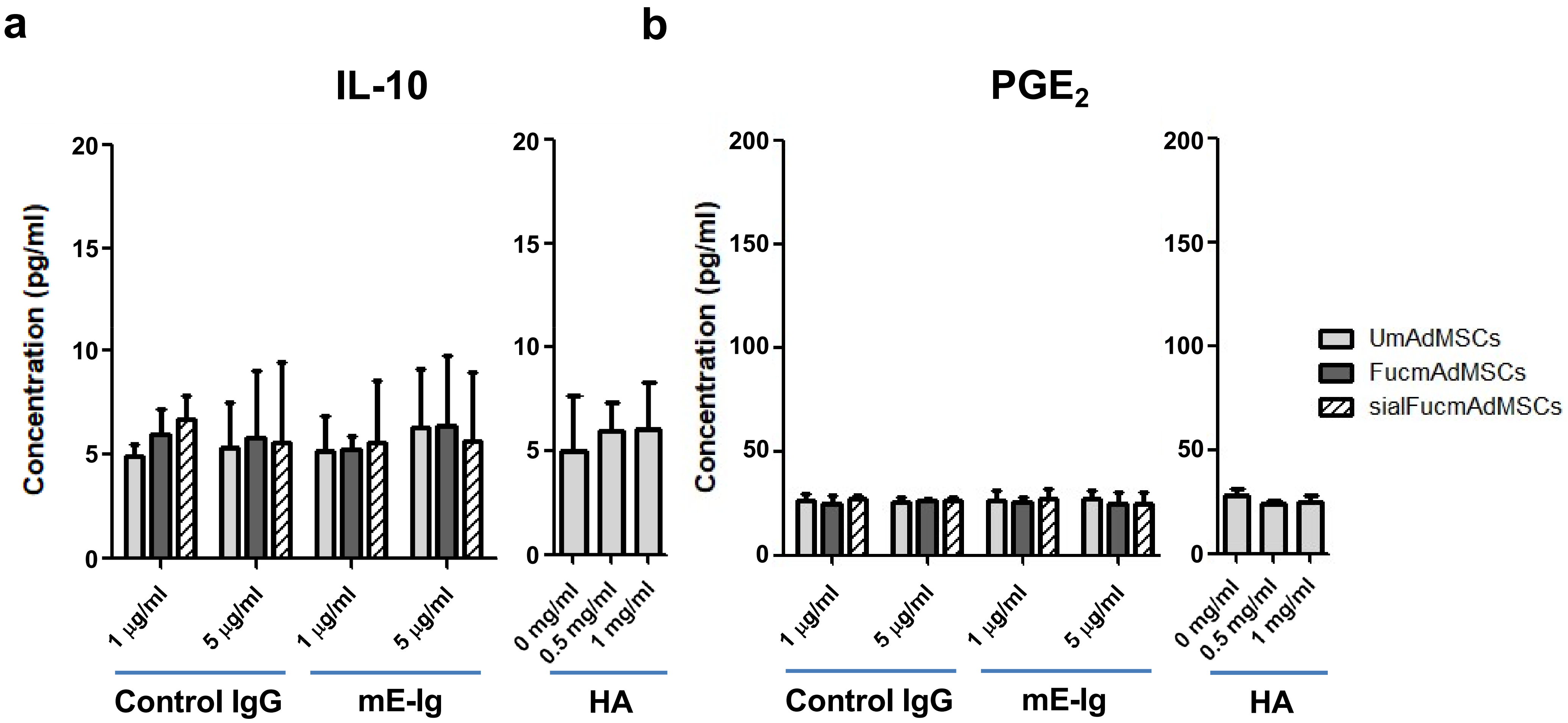

Supplementary Fig. 2 Culture supernatant levels of IL-10 and PGE_2 in mAdMSCs after HCELL or CD44 ligation. UmAdMSCs or FucmAdMSCs were cultured in the presence of different concentrations of murine E- selectin (mE-Ig) or hyaluronic acid (HA) at 37ºC for 24h, and culture supernatants were collected. **a** Interleukin-10 (IL-10), or **b** prostaglandin E_2 (PGE_2) levels, were measured by ELISA. Cells cultured in the absence of HA or in presence of control IgG served as negative controls. Also, as controls to assess specificity of E-selectin binding, FucmAdMSCs were treated with sialidase (sialFucmAdMSCs) to cleave terminal sialic acid from sLe^x. Concentrations levels of both factors are presented as mean ± SD for n=3 separate experiments, each performed in triplicates and statistically analyzed by one-way ANOVA with Tukey's multiple-comparisons test.

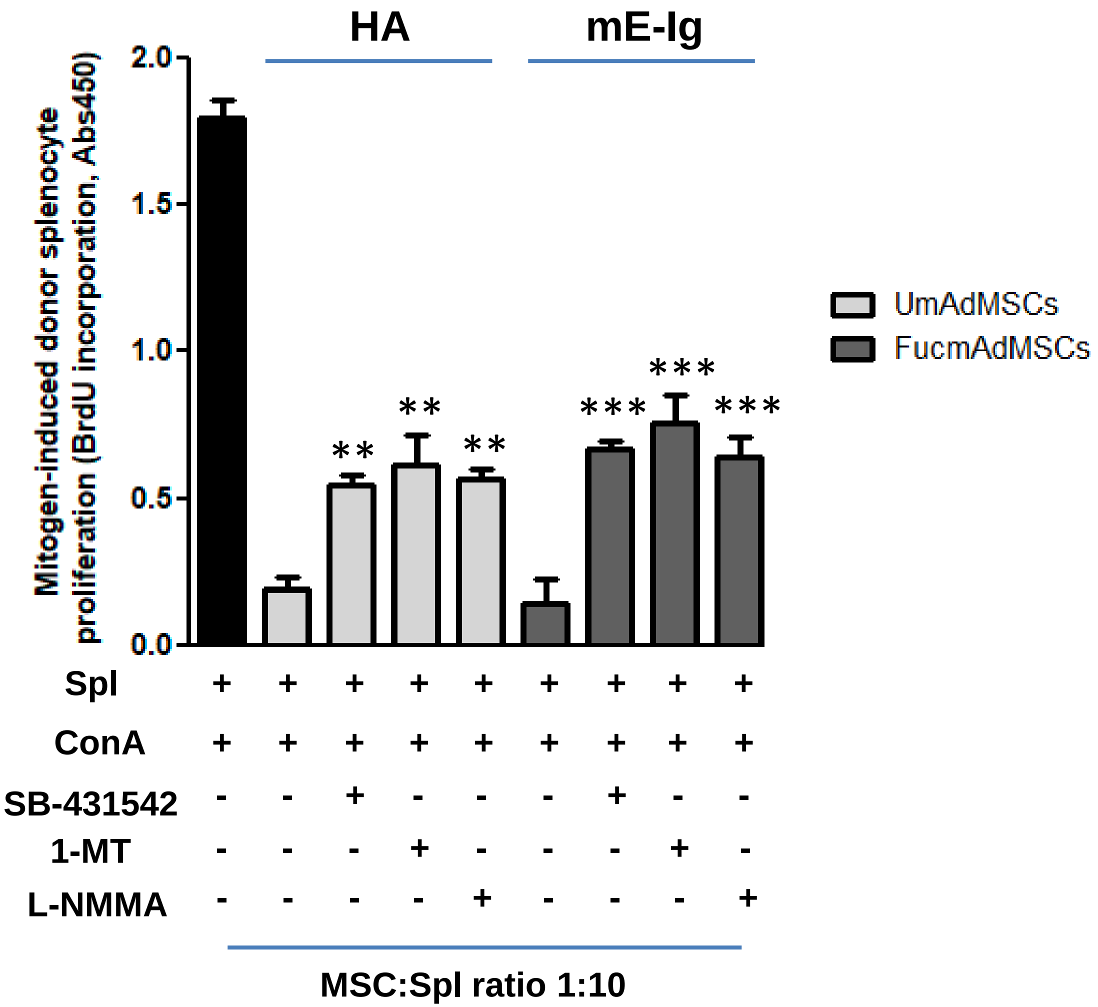

Supplementary Fig. 3 Inhibition of TGFβ signaling, or of production of IDO and NO, attenuates mAdMSC suppression of mitogen-induced splenocyte proliferation. Co-cultivation of C57BL/6 UmAdMSCs or FucmAdMSCs with BALB/c splenocytes was performed at a relatively high MSC:splenocyte ratio (1:10) in presence of concanavalin A (ConA), with or without co-incubation with hyaluronic acid (HA) or murine E-selectin (mE-Ig). Mitogen-induced splenocyte proliferation was calculated by subtracting the level of splenocyte basal proliferation in the absence of ConA. This assay was undertaken to assess whether the suppression of splenocyte proliferation mediated by MSC production of anti-inflammatory molecules would persist despite an increased MSC:splenocyte contact ratio (1:10). As shown, culture addition of SB-431542 (TGFβ signaling inhibitor), 1-methyl-DL-tryptophan (1-MT) (IDO inhibitor) or N^G-monomethyl-L- arginine (L-NMMA) (iNOS inhibitor) in each case significantly increases the proliferation of responder splenocytes compared to controls, ***p*<0.01 or ****p*<0.001, respectively. All data are presented as the mean ± SD of n=3 separate experiments and statistically analyzed by one-way ANOVA with Tukey's multiple-comparisons test.

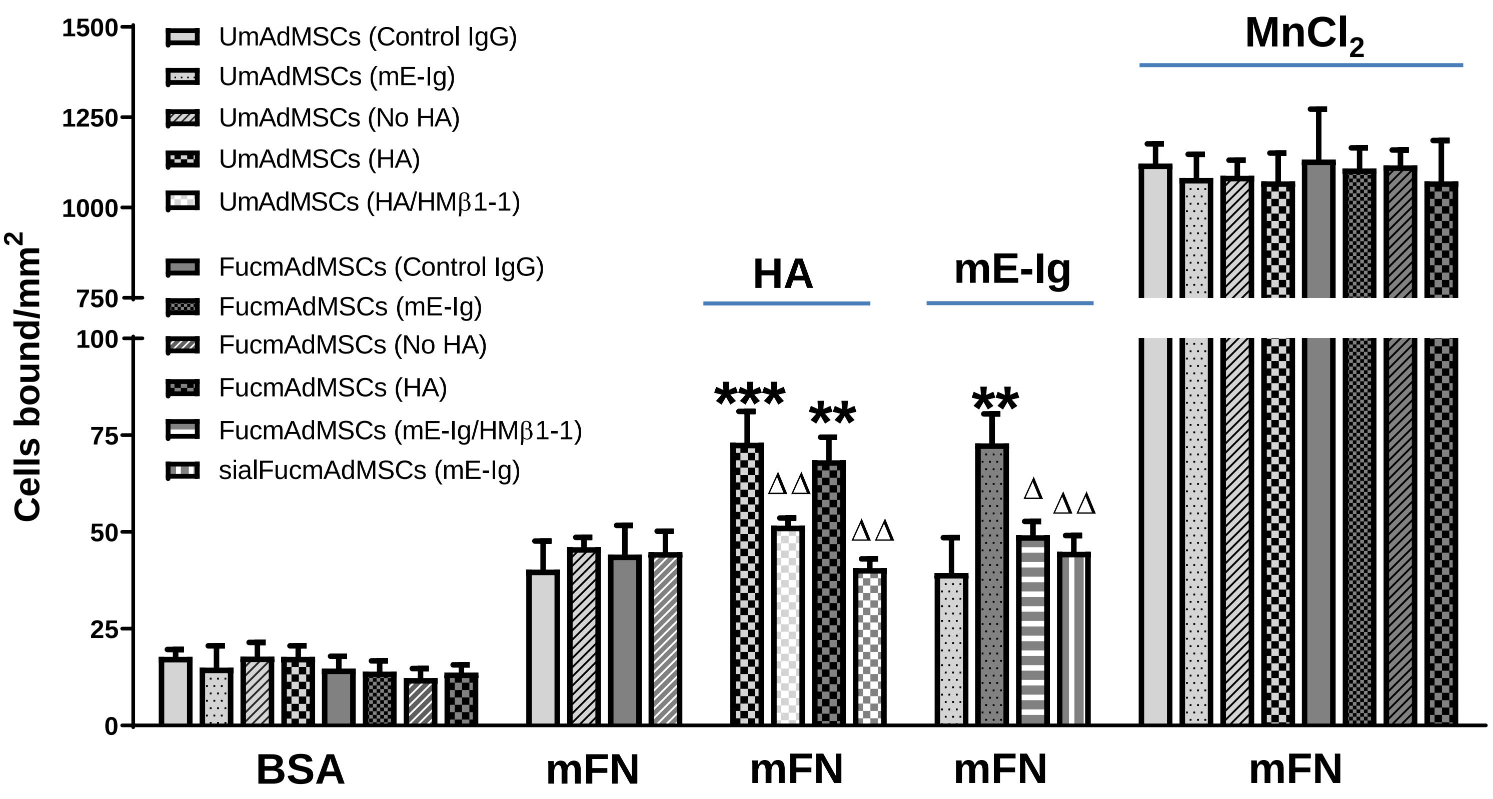

Supplementary Fig. 4 Effect of HCELL binding to E-selectin and CD44 binding to hyaluronic acid on β1 integrin-mediated adhesion of mAdMSCs to fibronectin. To evaluate UmAdMSCs or FucmAdMSCs adherence to fibronectin, baseline mAdMSC binding to BSA (negative control) and to mouse fibronectin (mFN) was quantified, then cells were pre-treated with E-selectin (mE-Ig, 5 μg/ml in HBSS medium containing 2 mM $CaCl_2$ and 0.2% BSA) or hyaluronic acid (HA, 1 mg/ml in DMEM medium containing 0.2% BSA) at 37ºC for 1h, and β1 integrin-mediated adhesion to mFN was then evaluated in the presence or absence of function-blocking anti-β1 antibody (clone HMβ1-1). Adhesion to mFN was significantly upregulated by ligation of HCELL or CD44 as shown (**p<0.01 or ***p<0.001). Treatment of FucmAdMSCs with sialidase (sialFucmAdMSCs), or treatment of UmAdMSCs or FucmAdMSCs with anti-β1 antibody HMβ1-1, in each case abrogated β1 integrin-mediated adhesion to mFN compared to respective levels found in UmAdMSCs or FucmAdMSCs previously exposed to E-selectin or HA ($^{\Delta}p$<0.05, $^{\Delta\Delta}p$<0.01). As positive control baseline to assess whether fucosylation and/or other cell manipulation(s) have direct effects on UmAdMSC or FucmAdMSC interactions with mFN, β1 integrins were maximally stimulated by exposure to 1 mM $MnCl_2$. All data are presented as the mean ± SD of n=3 separate experiments and statistically analyzed by one-way ANOVA with Tukey's multiple-comparisons test.
