# Supplementary material for: Enforced mesenchymal stem cell tissue colonization counteracts immunopathology
Source: NPJ Regen Med. 2022 Oct 19;7:61. doi: 10.1038/s41536-022-00258-z (PMC9582223; doi:10.1038/s41536-022-00258-z)
Supplement: Supplementary file 2 — REPORTING SUMMARY [file 41536_2022_258_MOESM2_ESM.pdf]

nature research

Corresponding author(s): Robert Sackstein

Last updated by author(s): October 01, 2022

Reporting Summary

Nature Research wishes to improve the reproducibility of the work that we publish. This form provides structure for consistency and transparency in reporting. For further information on Nature Research policies, see Authors & Referees and the Editorial Policy Checklist.

Statistics

For all statistical analyses, confirm that the following items are present in the figure legend, table legend, main text, or Methods section.

n/a	Confirmed	
☐	☒	The exact sample size (n) for each experimental group/condition, given as a discrete number and unit of measurement
☐	☒	A statement on whether measurements were taken from distinct samples or whether the same sample was measured repeatedly
☐	☒	The statistical test(s) used AND whether they are one- or two-sided<br>*Only common tests should be described solely by name; describe more complex techniques in the Methods section.*
☒	☐	A description of all covariates tested
☐	☒	A description of any assumptions or corrections, such as tests of normality and adjustment for multiple comparisons
☐	☒	A full description of the statistical parameters including central tendency (e.g. means) or other basic estimates (e.g. regression coefficient) AND variation (e.g. standard deviation) or associated estimates of uncertainty (e.g. confidence intervals)
☐	☒	For null hypothesis testing, the test statistic (e.g. F, t, r) with confidence intervals, effect sizes, degrees of freedom and P value noted<br>*Give P values as exact values whenever suitable.*
☒	☐	For Bayesian analysis, information on the choice of priors and Markov chain Monte Carlo settings
☒	☐	For hierarchical and complex designs, identification of the appropriate level for tests and full reporting of outcomes
☐	☒	Estimates of effect sizes (e.g. Cohen's d, Pearson's r), indicating how they were calculated

Our web collection on statistics for biologists contains articles on many of the points above.

Software and code

Policy information about availability of computer code

Data collection	Zeiss Axio Vision v.4.8 and BD FACSDiva 6.1.
Data analysis	GraphPad Prism v.5.0

For manuscripts utilizing custom algorithms or software that are central to the research but not yet described in published literature, software must be made available to editors/reviewers. We strongly encourage code deposition in a community repository (e.g. GitHub). See the Nature Research guidelines for submitting code & software for further information.

Data

Policy information about availability of data

All manuscripts must include a data availability statement. This statement should provide the following information, where applicable:
- Accession codes, unique identifiers, or web links for publicly available datasets
- A list of figures that have associated raw data
- A description of any restrictions on data availability

All data supporting the findings in this study are available from the corresponding authors on reasonable request

Field-specific reporting

Please select the one below that is the best fit for your research. If you are not sure, read the appropriate sections before making your selection.

☒ Life sciences ☐ Behavioural & social sciences ☐ Ecological, evolutionary & environmental sciences

For a reference copy of the document with all sections, see nature.com/documents/nr-reporting-summary-flat.pdf

Life sciences study design

All studies must disclose on these points even when the disclosure is negative.

Sample size	To calculate sample size in survival analysis (log-rank test) we define a type I error (alpha)= 0.05, a type error (beta)= 0.20, an hypothesized survival rate in group 1 (i.e FucmAdMSCs-treated-group)=0.8, an hypothesized survival rate in group 2 (i.e UmAdMSCs-treated group)=0.4, and a ratio of sample sizes in group 1/group 2= 1 (equal sample size in both groups), obtaining a total of n=27 animals/group. With only n=16 animals/group, a similar number of animals used in other previously reported studies using the same GvHD model, we obtain a p=0.0198 when compared survival of both treatment groups (FucAdMSCs vs UmAdMSCs-treated groups). Number of animals was not finally augmented in order to safeguard the animal welfare (3R principles). For all other experiments we used a minimum of n=3 replicates.
Data exclusions	No data were excluded from the analyses.
Replication	We confirmed the reproducibility of data by independently repeating all of the experiments at least three times. All attempts at replication were successful.
Randomization	Samples or animals were randomly allocated to the different experimental groups.
Blinding	Histopathological analyses were performed by a single pathologist blinded to the treatment groups using coded slices. Survival and GvHD clinical scores were also monitored by researchers in a blind fashion.

Reporting for specific materials, systems and methods

We require information from authors about some types of materials, experimental systems and methods used in many studies. Here, indicate whether each material, system or method listed is relevant to your study. If you are not sure if a list item applies to your research, read the appropriate section before selecting a response.

Materials & experimental systems

n/a	Involved in the study	
☐	☒	Antibodies
☐	☒	Eukaryotic cell lines
☒	☐	Palaeontology
☐	☒	Animals and other organisms
☒	☐	Human research participants
☒	☐	Clinical data

Methods

n/a	Involved in the study	
☒	☐	ChIP-seq
☐	☒	Flow cytometry
☒	☐	MRI-based neuroimaging

Antibodies

Antibodies used	We used specific antibodies for the following markers: CD44 (Supplier name: BD Biosciences; Cat.No: 550538; Clone: IM7; Lot number: 13122; Reactivity: mouse; Application: IHC-RT; Supplier name: Santa Cruz Biotechnology; Cat.No: sc-18882; Lot number: 051112; Reactivity: mouse, rat and human; Application: Immunofluorescence (IF), IHC (P) and FC); E-selectin (Supplier name: Abcam; Cat.No: ab18981; Clone: polyclonal; Lot number: GR190124; Reactivity: mouse, rat and human; Application: immunocytochemistry (ICC), IF, IHC, western blot (WB)); CD31 (Supplier name: Abcam; Cat.No: ab28364; Clone: polyclonal; Lot number: GR291622; Reactivity: mouse, human and pig; Application: IHC, ICC, WB); GFP (Supplier name: Aves Labs; Cat.No: GFP-1020; Clone: polyclonal; Lot number: 0511GP12; Reactivity: n/a; Application: IHC); CD3 (Supplier name: Dako; Cat.No: A045229-2; Clone: polyclonal; Lot number: 20047044; Reactivity: human and mouse (RL); Application: IHC, WB/ immunoprecipitation (IP), ELISA); CD29 (Supplier name: BioLegend; Cat.No: 102201; Clone: HMβ1-1; Reactivity: mouse/rat; Application: IP, IHQ).
Validation	Antibodies used for flow cytometry experiments were previously validated on murine AdMSCs, and the obtained percentages corresponded well to the expected values. Immunohistochemistry antibodies were also previously validated on appropriate fixed paraffin-embedded mouse tissue sections.

Eukaryotic cell lines

Policy information about cell lines

Cell line source(s)	Only primary murine adipose-derived mesenchymal stem cells (MSCs) and human bone marrow/adipose-derived MSCs were used in this study.
Authentication	Cells were authenticated following the minimal criteria for defining multipotent mesenchymal stem cells established by the International Society for Cellular Therapy.
Mycoplasma contamination	Murine and human MSCs were routinely tested for mycoplasma and murine or human pathogen contamination. No contamination was found at any time point during the course of the study.

Commonly misidentified lines (See ICLAC register)	No commonly misidentified cell lines were used.

Animals and other organisms

Policy information about studies involving animals; ARRIVE guidelines recommended for reporting animal research

Laboratory animals	For mAdMSCs isolation, we used C57BL/6 and C57BL/6-Tg(CAG-EGFP)(males and females, age: 8-10 weeks). At time of transplant, recipient C57BL/6 and donor BALB/c mice were age 10 and 12 weeks, and all experimental groups were composed by 50% males and 50% females.
Wild animals	This study did not involve wild animals.
Field-collected samples	This study did not involve field-collected samples.
Ethics oversight	All animal procedures were approved by the Institutional Animal Care and Use Committee at University of Murcia and performed according to the guidelines of our institution (approved protocol A13150201). The Institutional Review Board of the University Hospital Virgen de la Arrixaca (Murcia, Spain) approved the protocols used to obtain and process all human samples. As needed, written informed consent was obtained from donors as per Helsinki Declaration guidelines.

Note that full information on the approval of the study protocol must also be provided in the manuscript.

Flow Cytometry

Plots

Confirm that:

☐ The axis labels state the marker and fluorochrome used (e.g. CD4-FITC).

☐ The axis scales are clearly visible. Include numbers along axes only for bottom left plot of group (a 'group' is an analysis of identical markers).

☐ All plots are contour plots with outliers or pseudocolor plots.

☒ A numerical value for number of cells or percentage (with statistics) is provided.

Methodology

Sample preparation	Murine AdMSCs (mAdMSCs), human AdMSCs (hAdMSCs) and human BMMSCs (hBMMSCs) were isolated as described previously. In brief, mAdMSCs from C57BL/6 or C57BL/6-Tg (CAG-EGFP) mice, or hAdMSCs and hBMMSCs from healthy human donors, were flask-seeded in DMEM low glucose medium (Gibco, Carlsbad, CA, United States) supplemented with 15% fetal bovine serum (Gibco), 1% L-glutamine (Lonza, Basel, Switzerland), 100 U/ml penicillin, and 100 ug/ml streptomycin (Lonza) (complete medium). MSCs in culture passages 3-4 were used for experiments.
Instrument	*Identify the instrument used for data collection, specifying make and model number.*
Software	*Describe the software used to collect and analyze the flow cytometry data. For custom code that has been deposited into a community repository, provide accession details.*
Cell population abundance	*Describe the abundance of the relevant cell populations within post-sort fractions, providing details on the purity of the samples and how it was determined.*
Gating strategy	*Describe the gating strategy used for all relevant experiments, specifying the preliminary FSC/SSC gates of the starting cell population, indicating where boundaries between "positive" and "negative" staining cell populations are defined.*

☐ Tick this box to confirm that a figure exemplifying the gating strategy is provided in the Supplementary Information.
